# Trends in Intracranial Glioma Incidence and Mortality in the United States, 1975-2018

**DOI:** 10.3389/fonc.2021.748061

**Published:** 2021-11-01

**Authors:** Dongdong Lin, Ming Wang, Yan Chen, Jie Gong, Liang Chen, Xiaoyong Shi, Fujun Lan, Zhongliang Chen, Tao Xiong, Hu Sun, Shu Wan

**Affiliations:** Brain Center, Affiliated Zhejiang Hospital, Zhejiang University School of Medicine, Hangzhou, China

**Keywords:** glioma, glioblastoma, trends, age-adjusted incidence, incidence-based mortality, relative survival

## Abstract

**Purpose:**

Glioma incidence in the US seems to have stabilized over the past 20 years. It’s also not clear whether changes in glioblastoma incidence are associated with glioma mortality trends. Our study investigated trends in glioma incidence and mortality according to tumor characteristics.

**Methods:**

This study obtained data from the Surveillance, Epidemiology, and End Results-9 (SEER-9) registries to calculate glioma incidence and mortality trends. Annual percent changes (APC) and 95% CIs were calculated using the Joinpoint program.

**Results:**

62,159 patients (34,996 males and 55,424 whites) were diagnosed with glioma during 1975-2018, and 31,922 deaths occurred from 1995-2018. Glioblastoma (32,893 cases) and non-glioblastoma astrocytoma (17,406 cases) were the most common histologic types. During the study period, the incidence of glioma first experienced a significant increase (APC=1.8%, [95% CI, 1.3% to 2.3%]) from 1975 to 1987, and then experienced a slight decrease (APC=-0.4%, [95% CI, -0.5% to -0.3%]) from 1987 to 2018, while the APC was 0.8% for glioblastoma, -2.0% for non-glioblastoma astrocytoma, 1.1% for oligodendroglial tumors, 0.7% for ependymoma and -0.3% for glioma NOS during the study period. Glioblastoma incidence increased for all tumor size and tumor extension except for distant. From 1995 to 2018, glioma mortality declined 0.4% per year (95% CI: -0.6% to -0.2%) but only increased in patients older than 80 years [APC=1.0%, (95% CI, 0.4% to 1.6%)].

**Conclusion:**

Significant decline in glioma incidence (1987-2018) and mortality (1995-2018) were observed. Epidemiological changes in non-glioblastoma astrocytoma contributed the most to overall trends in glioma incidence and mortality. These findings can improve understanding of risk factors and guide the focus of glioma therapy.

## Introduction

According to the latest cancer statistics report (1), the United States is expected to have 23,890 cases diagnosed with brain and other nervous system tumors, and 18,020 deaths in 2020. It has become the leading cause of cancer death among males aged birth to 39 years and females aged birth to 19 years. Malignant glioma is a devastating type of brain and other nervous system tumors because of its high malignancy, extremely high mortality rate, and recurrence risk, as well as the huge burden on society and families (2). It is responsible for most deaths of patients with primary brain tumors. The incidence and mortality of glioma increase significantly with advancing age. With the aging trend of American society, the burden associated with glioma may become a huge challenge.

Although no population-based global epidemiological survey of glioma has been reported, the incidence of glioma varies geographically. The incidence of glioma in the American and northern European populations was higher than that in the Asian population, reaching about 2 times (3). Regions also showed different incidence trends. A Japanese study reported that the incidence rate of malignant glioma has risen significantly among the elderly from 1989 to 2008 (4), while CBTRUS reported that the incidence of glioma in people over 40 years old in the US was relatively stable from 2000 to 2016 (2). Besides, glioma incidence varies significantly by age, sex, race (5), and other factors. Compared with the relatively low incidence of glioma, the annual number of deaths caused by glioma deserves people’s attention. The mortality rate is a convincing indicator to reflect the progress against glioma. However, few studied have focused on the mortality of glioma.

Because glioblastoma accounts for the majority of glioma (57.3%) and its 5-year survival rate is only 6.8% after diagnosed (1), it provides an impression that changes in its epidemiological data may reflect the incidence and mortality of glioma. To clarify this issue, the current study quantified the differences in glioma incidence and mortality trends based on demographic and tumor characteristics, and further compared them with glioblastoma.

## Methods

### Data Sources

Information on glioma incidence was extracted from the SEER-9 incidence database (6), containing cases from nine high-quality registries (San Francisco, Connecticut, Detroit, Hawaii, Iowa, New Mexico, Seattle, Utah, and Atlanta), which covers approximately 9.4% of the U.S. population. Though the SEER database contains cases diagnosed starting with 1973, the Seattle and Atlanta registries joined the SEER program in 1974 and 1975, respectively. So, we calculated incidence rates for cases diagnosed between 1975-2018, the only years for which all 9 registries have cases in the database.

The SEER-9 incidence-based mortality database (7) provides a convenient, intuitive mechanism to analyze glioma mortality derived from information recorded on death certificates. Differ from general mortality rates, the incidence-based mortality rates allow for a breakdown of mortality rates by variables associated with cancer onset. In our study, we calculated incidence-based mortality rates for patients who died during 1995-2018 and were diagnosed during 1975-2018 to guarantee as many deaths as possible and to reduce underestimation of early incidence-based mortality rates.

Relative survival was analyzed using the SEER-9 incidence database (6). It is defined as the ratio of observed survivors (all causes of death) in a group of cancer patients to the expected survivors in a group of non-cancer patients (8). One- to ten-years relative survival rates for glioma and glioblastoma diagnosed in different years were compared.

### Patients’ Characteristics

Patients’ Characteristics of this study included demographic and tumor characteristics, obtained from medical records and death certificates responsibly. Demographic characteristics used in this study were sex, race, age groups at diagnosis/death (younger than 20, 20-39, 40-59, 60-79, and 80 years or older), median household income ($/year) and rural-urban distribution.

Histologic types were classified according to codes of International Classification of Diseases for Oncology, Third Edition (ICD-O-3), and were grouped into glioblastoma, non-glioblastoma astrocytoma, oligodendroglial tumors, ependymoma, and gliomas NOS (**eTable 1** in the Supplement data) following the classification criterion of CBTRUS (the Central Brain Tumor Registry of the United States). Only tumors located in the brain (ICD-O-3 topography code C71) and malignant behavior (ICD-O-3 behavior code of/3) were included. Due to the lack of WHO grade in the SEER database, we defined glioma WHO grade according to the 2007 WHO Classification of Tumours of the Central Nervous System (9) (**eTable 2** in the Supplement data).

Four different sets of codes from 1983: Extent of Disease-4 codes (EOD-4) for 1983-1987, Extent of Disease-10 codes (EOD-10) for 1988-2003, Collaborative Staging codes (CS) for 2004-2015 and Tumor Size Summary for 2016+, were merged to define tumor extension and size. Tumor extension was divided into localized, regional, distant, and unknown stages.

### Data Analysis

Glioma incidence and mortality rates were generated by SEER*Stat version 8.3.9 and reported as per 100,000 person-years. All rates were age-adjusted to the 2000 US standard population and rate ratios were calculated based on these standardized rates. Rate ratios were used to assess the degree of change for glioma with histologic type, tumor extension, or size, as well as the impact of unknown variables.

Joinpoint Regression Program, version 4.7.0.0, was used to evaluate the trends of incidence and mortality and calculated annual percentage change (APC) and 95% CIs. This program takes trend data and fits the simplest joinpoint model that the data allow. It tests that an apparent change in trend is statistically significant using a Monte Carlo Permutation method (10).

A two-sided P value of 0.05 was considered statistically significant.

## Results

During 1975-2018, 62,159 cases diagnosed as glioma in the states recorded by SEER-9 were included in the incidence analysis (**Table 1**). Men (34,996[56.3%]) and white cases (55,424[89.2%]) constituted the principal part of the patients. Among them, glioblastoma (32,893[52.9%]) and non-glioblastoma astrocytoma (17,406 [28.0%]) were the two most common histological types. The occurrence of glioma was mainly concentrated in the middle-aged and elderly population. Additionally, incidence-based mortality analysis revealed 31,922 patients with glioma died from 1995-2018. Of the deaths, 18,322 (57.4%) cases were men and 28,297 (88.6%) cases were white patients. Among those who died, patients tended to be older, diagnosed with glioblastoma, and with a localized or larger tumor.

**Table 1:** Glioma Incidence (1975-2018) and Incidence-Based Mortality (1995-2018): the SEER-9 registry database.

Characteristic	Incidence				Incidence-Based Mortality			
	Glioma		Glioblastoma		Glioma		Glioblastoma	
	Cases,No. (%)	Rate (95% CI)^a^	Cases,No. (%)	Rate (95% CI)^a^	Deaths,No. (%)^b^	Rate (95% CI)^a^	Deaths,No. (%)^b^	Rate (95% CI)^a^
Overall	62159	5.60 (5.56-5.65)	32986	2.99 (2.96-3.02)	31922	4.59 (4.54-4.64)	21201	3.04 (3.00-3.08)
Sex								
Male	34996 (56.3)	6.78 (6.71-6.85)	19009 (57.6)	3.80 (3.78-3.85)	18322 (57.4)	5.74 (5.65-5.82)	12281 (57.9)	3.87 (3.80-3.94)
Female	27163 (437)	4.61 (4.56-4.67)	13977 (42.4)	2.33 (2.29-2.37)	13600 (42.6)	3.63 (3.57-3.69)	8920 (42.1)	2.35 (2.30-2.40)
Race								
White	55424 (89.2)	6.22 (6.17-6.28)	29920 (90.5)	3.32 (3.28-3.36)	28297 (88.6)	5.15 (5.09-5.21)	18992 (89.6)	3.42 (3.37-3.47)
Black	3646 (5.9)	3.25 (3.14-3.36)	1727 (5.2)	1.64 (1.56-1.73)	1858 (5.8)	2.68 (2.56-2.81)	1123 (5.3)	1.71 (1.61-1.82)
Other^c^	3089 (5.0)	2.79 (2.69-2.89)	1426 (4.3)	1.36 (1.29-1.44)	1767 (5.5)	2.24 (2.14-2.35)	1086 (5.1)	1.40 (1.32-1.49)
Age at Diagnosis/Death, y								
<20	6099 (9.8)	1.90 (1.86-1.95)	434 (1.3)	0.14 (0.12-0.15)	926 (2.9)	0.51 (0.47-0.54)	188 (0.9)	0.10 (0.09-0.12)
20-39	9035 (14.5)	2.68 (2.62-2.74)	1793 (5.4)	0.55 (0.52-0.57)	2284 (7.2)	1.21 (1.16-1.26)	724 (3.4)	0.39 (0.36-0.42)
40-59	18163 (29.2)	6.34 (6.24-6.43)	10231 (31.0)	3.50 (3.44-3.57)	9642 (30.2)	5.05 (4.95-5.15)	5968 (28.1)	3.08 (3.00-3.16)
60-79	23987 (38.6)	16.21 (16.00-16.42)	17062 (51.7)	11.56 (11.39-11.74)	14989 (47.0)	16.47 (16.21-16.74)	11336 (53.5)	12.49 (12.26-12.73)
≥80	4875 (7.8)	14.42 (14.00-14.83)	3466 (10.5)	10.25 (9.92-10.60)	4081 (12.8)	17.81 (17.26-18.36)	2985 (14.1)	13.05 (12.59-13.53)
Median Household Income, $/year (1990-2018)								
<75000	25019 (55.2)	5.62 (5.55-5.69)	14098 (54.8)	3.14 (3.09-3.19)	17002 (55.4)	2.44 (2.41-2.48)	11615 (55.0)	1.66 (1.63-1.69)
≥75000	20307 (44.8)	5.62 (5.55-5.70)	11650 (45.2)	3.21 (3.15-3.27)	13710 (44.6)	1.97 (1.94-2.01)	9513 (45.0)	1.36 (1.34-1.39)
Rural-Urban Distribution (1990-2018)								
Urban	39043 (86.9)	5.64 (5.59-5.70)	22116 (86.6)	3.20 (3.16-3.24)	26328 (86.4)	3.79 (3.74-3.83)	18186 (86.6)	2.60 (2.57-2.64)
Rural	5867 (13.1)	5.68 (5.53-5.83)	3431 (13.4)	3.13 (3.02-3.24)	4130 (13.6)	0.60 (0.58-0.62)	2808 (13.4)	0.40 (0.39-0.42)
Histologic Type								
Glioblastoma	32893 (52.9)	2.98 (2.95-3.01)			21123 (66.2)	3.03 (2.99-3.07)		
Non-glioblastoma astrocytoma	17406 (28.0)	1.55 (1.53-1.58)			5803 (18.2)	0.84 (0.82-0.86)		
Oligodendroglial tumors	5788 (9.3)	0.52 (0.51-0.53)			2641 (8.3)	0.38 (0.37-0.39)		
Ependymoma	1474 (2.4)	0.13 (0.12-0.14)			453 (1.4)	0.07 (0.06-0.07)		
Glioma, NOS	4598 (7.4)	0.42 (0.41-0.43)			1902 (6.0)	0.28 (0.27-0.29)		
WHO Grade								
I	NR^d^	NR^d^			NR^d^	NR^d^		
II	19292 (31.0)	1.72 (1.70-1.74)			6016 (18.8)	0.87 (0.85-0.89)		
III	5242 (8.4)	0.47 (0.46-0.48)			2785 (8.7)	0.40 (0.39-0.42)		
IV	32893 (52.9)	2.98 (2.95-3.01)			21123 (66.2)	3.03 (2.99-3.07)		
Unknown	4729 (7.6)	0.43 (0.42-0.44)			1997 (6.3)	0.29 (0.28-0.31)		
Tumor Extension (1983-2015)								
Localized	37142 (76.2)	4.35 (4.30-4.39)	19674 (75.6)	2.32 (2.29-2.35)	20187 (75.2)	3.42 (3.37-3.47)	13498 (75.8)	2.29 (2.25-2.33)
Regional	8089 (16.6)	0.95 (0.93-0.97)	4623 (17.8)	0.54 (0.53-0.56)	4984 (18.6)	0.84 (0.82-0.87)	3349 (18.8)	0.57 (0.55-0.59)
Distant	208 (0.4)	0.02 (0.02-0.03)	104 (0.4)	0.01 (0.01-0.02)	85 (0.3)	0.02 (0.01-0.02)	54 (0.3)	0.01 (0.01-0.01)
Unknown	3299 (6.8)	0.39 (0.38-0.41)	1607 (6.2)	0.19 (0.18-0.20)	1605 (6.0)	0.28 (0.26-0.29)	918 (5.2)	0.16 (0.15-0.17)
Tumor size, cm (1983-2018)								
≤3.0	8972 (16.6)	0.94 (0.92-0.96)	4584 (15.6)	0.48 (0.47-0.49)	5152 (16.3)	0.74 (0.72-0.76)	3495 (16.5)	0.50 (0.48-0.52)
>3.0 to ≤5.0	13761 (25.4)	1.44 (1.42-1.46)	8813 (29.9)	0.92 (0.90-0.94)	9225 (29.3)	1.33 (1.30-1.35)	7019 (33.1)	1.01 (0.98-1.03)
>5.0	10160 (18.8)	1.06 (1.04-1.08)	6561 (22.3)	0.68 (0.67-0.70)	6975 (22.1)	1.00 (0.98-1.02)	5373 (25.4)	0.77 (0.75-0.79)
Unknown	21189 (39.2)	2.22 (2.19-2.25)	9488 (32.2)	1.00 (0.98-1.02)	10166 (32.3)	1.47 (1.44-1.50)	5290 (25.0)	0.76 (0.74-0.78)

a Rates were calculated as number of cases or deaths per 100,000 person-years and age-adjusted to the 2000 US standard population.

b Number of deaths were based on cases diagnosed during 1975-2018.

c Includes American Indian/Alaskan Native and Asian/Pacific Islander.

d Statistic suppressed because of fewer than 16 cases or deaths.

SEER, Surveillance, Epidemiology, and End Results; NOS, Not otherwise specified; WHO, World Health Organization.

Among all glioma patients in the study, we were unable to obtain information on tumor extension in approximately 21.6% of patients who were diagnosed before 1983 and after 2015 and the proportion in glioblastoma was 21.2%. There were also 13.0% of glioma patients and 10.7% of glioblastoma patients lacking tumor size information because the diagnosis was before 1983. Of the deaths during 1995-2018, 15.9% of glioma patients were diagnosed before 1983 and after 2015 with no information on tumor extension and the proportion in glioblastoma was 16%. 1.3% of glioma deaths and 0.1% of glioblastoma deaths were diagnosed before 1983 and lack of tumor size information. In the years that we can get information about tumor characteristics, WHO grade was unknown for 7.6% of glioma patients and 6.3% of deaths, tumor extension was unknown for 6.8% of glioma patients and 6.0% for deaths, and tumor size was unknown for 39.2% of glioma patients and 32.3% of deaths.

**Figure 1:**
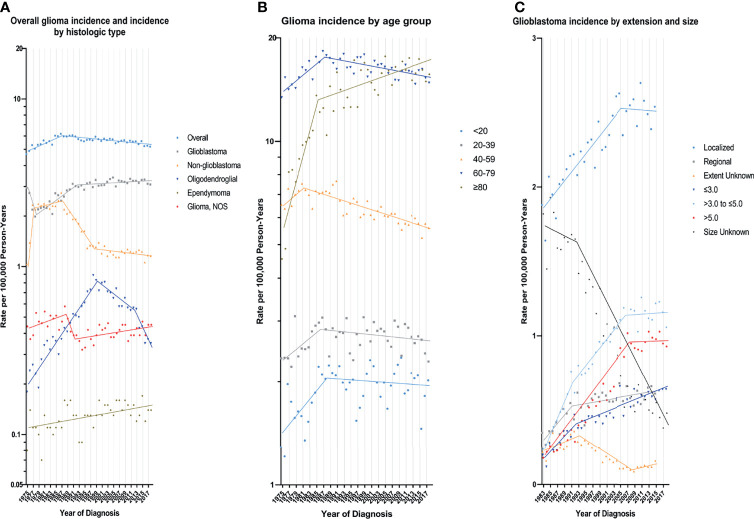
Trends in Annual Glioma Incidence Rates. **(A)** represents glioma incidence (1975-2018), overall and by histologic type. **(B)** represents glioblastoma incidence (1975-2018) by age group. **(C)** represents glioblastoma incidence by tumor extension (1983-2015) and size (1983-2018). Each point represents the observed incidence rates (100,000 person-years). The slope of the lines represents the annual percent change (APC). All rates are age-adjusted to the 2000 US standard population.

According to demographic and glioma characteristics, trends in glioma incidence was showed in **Figure 1** and **Table 2**. Joinpoint program divided the trends into a minimum of 1 to a maximum of 5. During the entire study period, glioma incidence did not change significantly (APC=0.0), but its incidence increased significantly from 1975 to 1987 (APC=1.8%, [95% CI, 1.3% to 2.3%]), and decreased significantly from 1987 to 2018 (APC=-0.4%, [95% CI, -0.5% to -0.3%]). Glioma incidence in male was 1.47 times higher than that in female, and the difference increased with age (from 1.08 [95% CI: 1.02 to 1.13] times in those younger than 20 to 1.67 [95% CI: 1.58 to 1.77] times in those older than 80) (**eTable 3** in the Supplement data). The difference in glioblastoma incidence between male and female was more significant. But both incidence trends were similar to the overall trend. Glioma incidence increased for whites, patients younger than 20 years or older than 80 years, while it decreased in the population aged 40-59 years. From 1990 to 2018, there were significantly decreasing incidence trends in both high- or low-income families and urban or rural patients. In terms of glioma histology, positive trends were observed for glioblastoma (APC=0.8%, [95% CI, 0.6% to 1.0%]), oligodendroglial tumor (APC=1.1%, [95% CI, 0.2% to 2.1%]) and ependymoma (APC=0.7%, [95% CI, 0.3% to 1.1%]), while non-glioblastoma astrocytoma (APC=-2.0%, [95% CI, -2.4% to -1.6%]) showed downward trends. Glioblastoma incidence increased by 2.9% (95% CI, 2.1% to 3.8%) per year from 1978 to 1992, but it slowed down during 1992-2018 (APC=0.2%, [95% CI, 0.0% to 0.5%]). Glioblastoma incidence increased significantly for all tumor size and extension groups except that the number of patients with distant metastases was too rare to reach statistical scope.

**Table 2:** Trends in Glioma Incidence Rates^a^ (1975-2018): the SEER-9 registry database.

Characteristic	Overall (1975-2018)		Trends^b^														
			1			2			3			4			5		
	APC (95%CI)	P Value	Year	APC (95%CI)	P Value	Year	APC (96%CI)	P Value	Year	APC (97%CI)	P Value	Year	APC (98%CI)	P Value	Year	APC (98%CI)	P Value
Overall	0.0 (-0.2 to 0.1)	0.581	1975-1987	1.8 (1.3 to 2.3)	<.001	1987-2018	-0.4 (-0.5 to -0.3)	<.001									
Sex																	
Male	-0.1 (-0.2 to 0.0)	0.205	1975-1987	1.6 (0.9 to 2.3)	<.001	1987-2018	-0.4 (-0.6 to -0.3)	<.001									
Female	0.0 (-0.2 to 0.2)	0.988	1975-1986	2.1 (1.3 to 3.0)	<.001	1986-2013	-0.3 (-0.4 to -0.1)	0.007	2013-2018	-1.8 (-3.8 to 0.3)	0.090						
Race																	
White	0.1 (0.0 to 0.3)	0.034	1975-1987	1.9 (1.4 to 2.5)	<.001	1987-2018	-0.2 (-0.3 to -0.1)	0.001									
Black	0.0 (-0.3 to 0.3)	0.825															
Other^c^	0.3 (0.0 to 0.6)	0.041	1975-1995	2.0 (1.0 to 3.1)	<.001	1995-2018	-0.5 (-0.9 to 0.0)	0.054									
Age at Diagnosis, y																	
<20	0.4 (0.1 to 0.8)	0.014	1975-1988	2.9 (0.9 to 4.9)	0.005	1988-2018	-0.2 (-0.7 to 0.3)	0.477									
20-39	0.1 (-0.2 to 0.3)	0.662	1975-1986	1.9 (0.0 to 3.8)	0.047	1986-2018	-0.2 (-0.6 to 0.1)	0.127									
40-59	-0.6 (-0.7 to -0.5)	<.001	1975-1981	2.0 (-0.7 to 4.8)	0.145	1981-2018	-0.7 (-0.9 to -0.6)	<.001									
60-79	-0.1 (-0.2 to 0.1)	0.444	1975-1987	1.9 (1.1 to 2.8)	<.001	1987-2018	-0.4 (-0.6 to -0.3)	<.001									
≥80	1.4 (1.0 to 1.8)	<.001	1975-1985	9.0 (4.2 to 14.0)	<.001	1985-2018	0.8 (0.4 to 1.2)	<.001									
Median Household Income (1990-2018)																	
<75000	-0.3 (-0.5 to -0.2)	<.001															
≥75000	-0.4 (-0.5 to -0.2)	<.001															
Rural-Urban (1990-2018)																	
Urban	-0.5 (-0.6 to -0.3)	<.001															
Rural	-0.4 (-0.7 to 0.0)	0.031															
Histologic Type																	
Glioblastoma	0.8 (0.6 to 1.0)	<.001	1975-1978	-11.9 (-18.9 to -4.3)	0.004	1978-1992	2.9 (2.1 to 3.8)	<.001	1992-2018	0.2 (0.0 to 0.5)	0.038						
Non-glioblastoma astrocytoma	-2.0 (-2.4 to -1.6)	<.001	1975-1977	49.8 (23.3 to 82.0)	<.001	1977-1987	1.1 (-0.2 to 2.3)	0.092	1987-1998	-5.8 (-6.8 to -4.7)	<.001	1998-2018	-0.5 (-0.9 to -0.1)	0.024			
Oligodendroglial tumors	1.1 (0.2 to 2.1)	0.020	1975-1999	6.1 (5.3 to 6.8)	<.001	1999-2012	-2.9 (-4.2 to -1.7)	<.001	2012-2018	-8.3 (-12.4 to -4.1)	<.001						
Ependymoma	0.7 (0.3 to 1.1)	0.001															
Glioma, NOS	-0.3 (-0.6 to -0.0)	0.057	1975-1988	1.5 (-0.1 to 3.1)	0.063	1988-1991	-10.8 (-33.0 to 18.9)	0.427	1991-2018	0.6 (0.1 to 1.1)	0.016						
WHO Grade																	
I	NR^d^																
II	-2.1 (-2.4 to -1.7)	<.001	1975-1977	42.1 (18.6 to 70.2)	<.001	1977-1987	0.6 (-0.6 to 1.8)	0.353	1987-1992	-6.3 (-10.1 to -2.2)	0.004	1992-2011	-1.7 (-2.1 to -1.2)	<.001	2011-2018	-5.7 (-7.7 to -3.6)	<.001
III	2.2 (1.5 to 2.9)	<.001	1975-1990	12.1 (8.9 to 15.4)	<.001	1990-2008	-0.4 (-1.7 to 1.0)	0.594	2008-2018	3.5 (0.9 to 6.2)	0.009						
IV	0.8 (0.6 to 1.0)	<.001	1975-1978	-11.9 (-18.9 to -4.3)	0.004	1978-1992	2.9 (2.1 to 3.8)	<.001	1992-2018	0.2 (0.0 to 0.5)	0.038						
Unknown	-0.2 (-0.5 to 0.1)	0.125	1975-1988	1.4 (-0.2 to 3.0)	0.076	1988-1991	-9.9 (-31.9 to 19.3)	0.458	1991-2018	0.6 (0.1 to 1.1)	0.014						
Glioblastoma																	
Tumor Extension (1983-2015)																	
Localized	1.0 (0.8 to 1.2)	<.001	1983-2005	1.4 (1.0 to 1.8)	<.001	2005-2015	-0.1 (-1.0 to 0.9)	0.857									
Regional	1.5 (1.0 to 2.0)	<.001	1983-1991	7.4 (2.8 to 12.2)	0.002	1991-2015	0.7 (0.1 to 1.3)	0.020									
Distant	NR^d^																
Unknown	-3.3 (-4.2 to -2.3)	<.001	1983-1993	3.9 (0.5 to 7.4)	0.027	1993-2008	-7.5 (-9.6 to -5.4)	<.001	2008-2015	5.0 (-2.0 to 12.6)	0.158						
Tumor size, cm (1983-2018)																	
≤3.0	2.6 (2.0 to 3.1)	<.001	1983-1992	9.3 (4.6 to 14.2)	<.001	1992-2018	1.8 (1.3 to 2.4)	<.001									
>3.0 to ≤5.0	2.8 (2.2 to 3.4)	<.001	1983-1991	14.0 (10.1 to 18.1)	<.001	1991-2006	3.4 (2.5 to 4.3)	<.001	2006-2018	0.1 (-0.8 to 1.0)	0.803						
>5.0	4.3 (3.6 to 5.0)	<.001	1983-2007	6.7 (6.1 to 7.4)	<.001	2007-2018	0.1 (-1.0 to 1.2)	0.805									
Unknown	-4.3 (-4.8 to -3.9)	<.001	1983-1992	-0.7 (-3.2 to 1.8)	0.566	1992-2018	-5.3 (-5.9 to -4.6)	<.001									

a Rates were calculated as number of cases or deaths per 100,000 person-years and age-adjusted to the 2000 US standard population.

b Each segment was defined by the joinpoint program when a statistically significant change in the APC occurred.

c Includes American Indian/Alaskan Native and Asian/Pacific Islander.

d APC could not be calculated because there were 0 case in one or more years.

SEER, Surveillance, Epidemiology, and End Results; APC, annual percent change; NOS, Not otherwise specified; WHO, World Health Organization.

**Figure 2:**
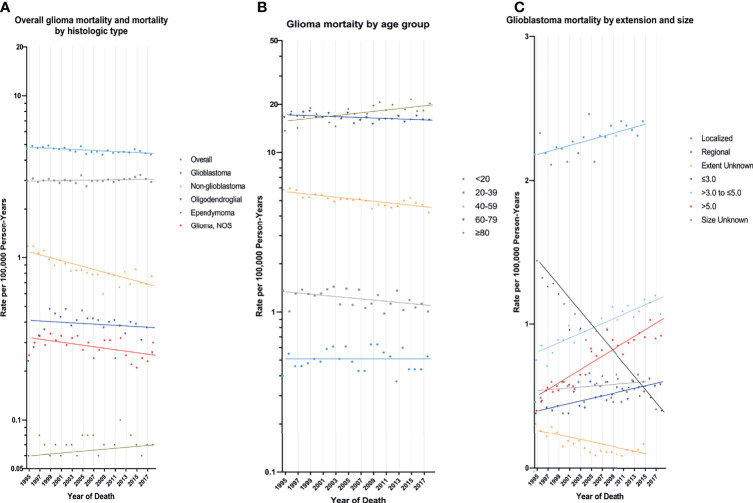
Trends in Annual Glioma Mortality Rates. **(A)** represents glioma mortality (1995-2018), overall and by histologic type based on cases diagnosed during 1975-2018. **(B)** represents glioblastoma mortality (1995-2018) by age group based on cases diagnosed during 1975-2018. **(C)** represents glioblastoma incidence by tumor extension (1995-2015) and size (1995-2018) based on cases diagnosed during 1983-2015 and 1983-2018, respectively. Each point represents the observed incidence-based mortality rates (100,000 person-years). The slope of the lines represents the annual percent change (APC). All rates are age-adjusted to the 2000 US standard population.

In the earliest years of the initial inclusion of cases, glioma incidence-based mortality rates may be underestimated. Therefore, we left a buffer time of 20 years to calculate the glioma mortality trends from 1995 to 2018 (**Figure 2** and **Table 3**). During this period, glioma incidence-based mortality rate decreased 0.4% (95% CI, -0.6% to -0.2%) per year averagely. Most demographic groups had negative APC with statistical difference, such as patients who were both genders, white, and diagnosed between 20 and 79 years old. It was worth noting that only patients older than 80 years showed a significant increase in incidence-based mortality (APC=1.0%, [95% CI, 0.4% to 1.6%]). There were also significant differences in mortality between male and female. The mortality rate of men was about 1.58 times than that of women, and among those over 80 years old, the proportion reached 1.67 (95% CI: 1.57 to 1.77) (**eTable 3** in the Supplement data). Interestingly, high-income patients showed a significant decrease in mortality (APC=-1.6%, [95% CI, -2.2% to -1.0%]), while low-income patients showed a significant increase in mortality (APC=1.1%, [95% CI, 0.6% to 1.6%]). By glioma histology, non-glioblastoma astrocytoma (APC=-2.1%, [95% CI, -2.6% to -1.5%]) and glioma NOS (APC=-1.0%, [95% CI, -1.8% to -0.2%]) exhibited significantly reduced mortality rates, while the mortality rates of glioblastoma, oligodendroglial tumor and ependymoma were relatively stable during this period. Additionally, only glioma patients with WHO grade II had a significant decrease (APC=-2.0%, [95% CI, -2.5% to -1.6%]) in incidence-based mortality among patients with known WHO grade. Significantly positive APC occurred for glioblastoma patients of localized tumor at diagnosis (APC=0.5%, [95% CI, 0.2% to 0.7%]). Glioblastomas of all known size showed significant increases in incidence-based mortality.

**Table 3:** Trends in Glioma Incidence-Based Mortality Rates^a^ (1995-2018): the SEER-9 registry database.

Characteristic	Overall (1995-2018)^b^	
	APC (95%CI)	P Value
Overall	-0.4 (-0.6 to -0.2)	<.001
Sex		
Male	-0.3 (-0.6 to -0.1)	0.018
Female	-0.5 (-0.8 to -0.2)	0.001
Race		
White	-0.2 (-0.4 to -0.1)	0.013
Black	0.0 (-0.6 to 0.7)	0.902
Other^c^	-0.1 (-0.8 to 0.6)	0.815
Age at Diagnosis/Death, y		
<20	0.0 (-0.9 to 1.0)	0.932
20-39	-0.9 (-1.5 to -0.2)	0.014
40-59	-1.0 (-1.3 to -0.7)	<.001
60-79	-0.3 (-0.6 to -0.1)	0.010
≥80	1.0 (0.4 to 1.6)	0.001
Median Household Income		
<75000	1.1 (0.6 to 1.6)	<.001
≥75000	-1.6 (-2.2 to -1.0)	<.001
Rural-Urban		
Urban	0.2 (0.0 to 0.4)	0.059
Rural	-1.4 (-1.9 to -0.9)	<.001
Histologic Type		
Glioblastoma	0.1 (-0.1 to 0.3)	0.188
Non-glioblastoma astrocytoma	-2.1 (-2.6 to -1.5)	<.001
Oligodendroglial tumors	-0.5 (-1.5 to 0.6)	0.391
Ependymoma	0.4 (-0.8 to 1.6)	0.527
Glioma, NOS	-1.0 (-1.8 to -0.2)	0.013
WHO Grade		
I	NR^d^	NR^d^
II	-2.0 (-2.5 to -1.6)	<.001
III	-0.3 (-1.2 to 0.6)	0.512
IV	0.1 (-0.1 to 0.3)	0.188
Unknown	-0.9 (-1.7 to -0.1)	0.038
Glioblastoma		
Tumor Extension^e^ (1995-2015)		
Localized	0.5 (0.2 to 0.7)	0.001
Regional	0.5 (-0.3 to 1.4)	0.191
Distant	NR^d^	NR^d^
Unknown	-4.6 (-6.2 to -2.9)	<.001
Tumor size^f^, cm		
≤3.0	1.8 (1.2 to 2.4)	<.001
>3.0 to ≤5.0	1.7 (1.3 to 2.1)	<.001
>5.0	3.1 (2.4 to 3.8)	<.001
Unknown	-5.5 (-6.1 to -4.9)	<.001

a Rates were calculated as number of deaths per 100,000 person-years and age-adjusted to the 2000 US standard population.

b Based on deaths during 1995-2018 and cases diagnosed during 1975-2018.

c Includes American Indian/Alaskan Native and Asian/Pacific Islander.

d APC could not be calculated because there were 0 death in one or more years.

e Tumor extension was based on cases diagnosed between 1983 and 2015.

f Tumor size was based on cases diagnosed between 1983 and 2018.

SEER, Surveillance, Epidemiology, and End Results; APC, annual percent change; NOS, Not otherwise specified; WHO, World Health Organization.

Survival analysis (**eTable 4** in the Supplement data) revealed that relative survival rates for glioma and glioblastoma increased with the year of diagnosis. The 1-year relative survival rate of glioma patients increased from 39.24% for cases diagnosed between 1975-1979 to 58.40% for cases diagnosed in 2017, while the 5-year relative survival rate increased from 14.84% for cases diagnosed between 1975-1979 to 27.25% for cases diagnosed in 2013. Besides, the 1-year relative survival rate of glioblastoma patients also increased from 26.18% for cases diagnosed between 1975-1979 to 44.90% for those diagnosed in 2017. Its 5-year relative survival rate peaked at 6.86% among the cases diagnosed in 2005 and has remained at a high level since then.

Rate Ratios were calculated for all glioma histologic types and glioblastoma by known and unknown variables of tumor extension and tumor size (**eTables 5** and **6** in the Supplement data). The incidence rate for glioblastoma with unknown tumor extension decreased from 1983-1987 to 2012-2015 by 0.54 (95% CI: 0.44 to 0.66) times, meaning that the observed increase in glioblastoma with known tumor extension was overestimated. Glioblastoma with unknown tumor size declined from 1983-1987 to 2013-2018 by 0.28 (95% CI: 0.25 to 0.30) times, indicating that the observed increase in glioblastoma with known tumor size was overestimated. Additionally, the mortality rate for glioblastoma with unknown tumor extension declined by 0.47 (95% CI: 0.39 to 0.57) times from 1995-1998 to 2011-2015, suggesting that glioblastoma deaths with known tumor extension were overestimated. The mortality rate for glioblastoma with unknown tumor size decreased by 0.35 (95% CI: 0.32 to 0.39) times from 1995-1998 to 2015-2018, meaning that the increase in the mortality rate for glioblastoma with known tumor size was overestimated.

**eTables 7**-**20** in the Supplement data shows the detailed number of cases, deaths, incidence rates, and incidence-based mortality rates per year.

## Discussion

The present study is the first to report intracranial glioma mortality trends in the United States. Incidence and mortality trends were compared systematically according to demographic and tumor characteristics. We also conducted a detailed analysis of the epidemiological trends for glioblastoma. The main finding was the significant decline in the age-adjusted incidence of glioma from 1987 to 2018 (APC=-0.4%, [95% CI, -0.5% to -0.3%]) and the incidence-based mortality from 1995 to 2018 (APC=-0.4%, [95% CI, -0.6% to -0.2%]). This finding is inconsistent with the significantly increased incidence and stable mortality of glioblastoma and appears to be related to the trends of non-glioblastoma astrocytoma.

The epidemiological studies of glioma published in recent years were presented in **Table 4**. There were differences in cancer registries, study years and design. According to the report, glioma incidence ranged from 4.80 to 7.70 per 100,000 person-years, while the incidence of glioblastoma ranged from 2.82 to 5.10 per 100,000 person-years. Glioma incidence of our study was 5.60 (5.56-5.65) per 100,000 person-years and that of glioblastoma was 2.99 (2.96-3.02) per 100,000 person-years. They were consistent with incidence reported in other US studies and lower than those reported in Nordic countries. However, there are few reports on glioma mortality.

**Table 4:** Incidence rate of glioma patients among countries.

Year of publication	Statistical years	Country	Data source	Incidence rate (per 100,000)	Tumor type	Authors
2021	2007-2017	Korea	Korean National Health Insurance Database (NHID)	7.47	glioma	Kim et al. (11)
2020	1995	England	Canadian Cancer Registry and Central Brain Tumor Registry of the United States (CBTRUS)	2.39	glioblastoma	Davis et al. (12)
2020	1995	Canada		3.56		
2020	1995	USA		3.92		
2020	2015	England		5.02		
2020	2015	Canada		4.50		
2020	2015	USA		4.32		
2019	2011-2015	USA-high SES	Central Brain Tumor Registry of the United States (CBTRUS)	5.88	glioma	Cote et al. (13)
2019	2011-2015	USA-low SES		5.52	glioma	
2019	2011-2015	USA-high SES		3.16	glioblastoma	
2019	2011-2015	USA-low SES		2.95	glioblastoma	
2019	1990-2006	Finland	Finnish Cancer Registry (FCR)	7.70	glioma	Natukka et al. (14)
2019	2007-2016			7.30		
2018	1973-2014	USA	SEER	6.90	glioma	Li et al. (15)
2018	1973-2014			4.10	glioblastoma	
2017	2009-2014	Denmark	Danish Neuro-Oncology Registry (DNOR)	7.30	glioma	Rasmussen et al. (16)
2017	2009-2014			5.10	glioblastoma	
2017	2013	Korea	Korean Central Cancer Registry	2.82	glioblastoma	Dho et al. (17)
2016	2001-2007	England-white	National Cancer Intelligence Network	4.80	glioma	Maile et al. (18)
2016	2005-2009	Canton of Zurich in Switzerland	Zurich and Zug Cancer Registry	3.90	glioblastoma	Gramatzki et al. (19)

SEER, Surveillance, Epidemiology, and End Results; SES, socioeconomic status.

Trends in the incidence rates are an effective measure for assessing the burden of disease in a specific population. We used the latest available data to analyze the 44-year incidence rates in parts of the United States. Over time, many factors could cause fluctuations in incidence rates. We strove to find significant trends in incidence over a certain period of this study and interpret the result. Joinpoint program analyzed that the incidence of glioma experienced a significant increase before 1987, and then began to slowly decline. The early increase in incidence was partly attributed to the improvement of diagnostic technology, especially the introduction of CT in the 1970s and MRI in the 1980s. The advancement and increasingly widespread use of imaging diagnostic technology may detect subtle or small brain glioma, and more patients with asymptomatic or only mild symptoms were found (20, 21). Our results also showed that the incidence of tumors (both glioma and glioblastoma) smaller than 3cm continued to increase throughout the study period. This factor may also contribute to the rapid increase in the incidence of glioblastoma by 2.9% per year from 1978 to 1992. Additionally, the early 9 SEER registries to improve cancer registration procedures may affect glioma incidence statistics.

With the popularization of glioma diagnosis methods, its rapidly increasing incidence rate began to decelerate and has continued to decline since 1987. However, the glioblastoma incidence trend was different, with a significant increase of 0.2% per year after 1992. Thus, changes in the use of cancer screening tests are not solely responsible for the incidence trends. Behaviors related to cancer risk are a small but actual factor that affects glioma incidence. Numerous studies have been conducted to investigate the environmental and behavioral risk factors for glioma. Only ionizing radiation is a well-validated factor that increases the risk of glioma (22). Two studies about children exposed to low-dose diagnostic radiation tests revealed suffering CT scans in childhood increases the risk of brain tumors in adulthood, but the incidence was extremely low, only one excess case of brain tumor per 10,000 head CT scans in the 10 years after the first CT scan (23, 24). Additional risk research found that patients receiving high-dose radiation therapy increase the risk of glioma (25). Yet, the concern is that medical radiation has indeed increased significantly (26) over the past 20 years, despite the clinical benefits outweigh the risks (27). Mobile phone use has also caused public health concerns about the risk of occurrence of glioma because the brain is the organ most affected by the radiofrequency field of mobile phone. Several epidemiological studies on mobile phone use and increased risk of glioma cannot reach valid conclusions (28, 29). In addition, occupational exposure and chemical agents have also been checked, with no evidence related to the risk of glioma (30, 31).

There is growing epidemiological evidence consistently suggesting that atopic diseases, such as allergies, asthma, eczema, etc. reduce the risk of glioma (32–35). Related research revealed that when the subject is in atopic conditions, the risk of glioma is reduced by 30-40% (36, 37). Interestingly, as the types of allergies and the duration of symptoms increase, the risk of glioma is further reduced (38). The underlying mechanism may be due to the overactive immune state that appears in the atopic conditions, which eliminates precancerous cells by increasing immune monitoring (39).

Several demographic characteristics and lifestyle-related factors are suspected of affecting the incidence of glioma. A study covering 99.9% of the U.S. population revealed that the incidence and survival rates of most glioma subtypes vary significantly depending on race or ethnicity, among which non-Hispanic whites have the highest incidence and the lowest survival rate (3, 5). The reason for this difference involves the variation in genetic risk susceptibility, access to health care, and socioeconomic status (SES). Another study based on CBTRUS data found glioma incidence in high SES counties was higher than in low SES counties (13). Ai Seon Kuan et al. summarized 3 large prospective studies and found that major food groups, nutrients, or common healthy dietary patterns were not associated with glioma incidence (40). Similar results have been observed in cigarette smoking and alcohol intake (41).

A significant decline in the mortality rate of glioma means that we have made certain achievements in the anti-tumor process during this period, even if it is affected to some extent by the decrease in incidence rate in recent years. Only those over 80 years of age have continued to experience an increase in mortality rate, possibly because of the increasing incidence of elderly patients as a result of an aging population. Furthermore, our results show that the significant decrease in the non-glioblastoma astrocytoma mortality rate contributed most to the overall mortality trend of glioma. Continuous improvement of diagnosis and treatment standards for patients with non-glioblastoma astrocytoma and precise management benefit low-grade glioma patients (42). As the incidence of glioblastoma continues to increase, the relatively stable mortality rates suggest that aggressive treatment methods, including surgery, radiotherapy, and chemotherapy, are effective for this highly malignant tumor. Especially since 2005, temozolomide has been widely used in the chemotherapy of glioblastoma. The survival rate of glioblastoma patients has been effectively improved (43). Our analysis of relative survival rates also supported this. It was found that the 5-year survival rate of glioblastoma patients increased obviously in 2004 and remained above 5% for a period of time thereafter. But the mortality rate did not show a significant downward trend around 2005. Tumor-treating fields (TTFields) is an emerging alternative treatment method, which combined with temozolomide chemotherapy can significantly prolong the survival of patients with glioblastoma (44). In addition, numerous clinical trials (45) of drugs targeting specific molecular markers for glioblastoma are underway, with no clear efficacy. The development of novel treatment sites and strategies are necessary.

We provide a comprehensive assessment of glioma incidence and mortality in the United States from 1975 to 2018 but must admit that there are several important limitations in this study. Due to the retrospective nature of this study, the accuracy of our results depends on the quality and availability of the SEER database. Individual-level risk factors, lifestyle habits, occupational exposure, and changes in glioma examination methods have not been recorded by registries. We can only speculate on possible explanations based on actual observed trends in glioma incidence and mortality. In addition, tumor extension and tumor size data were limited to the years in which the registries recorded, starting in 1983. And there were a large number of unknown values of these two indicators in the early recording period, and they decreased significantly with the years, especially the tumor size. It will lead us to overestimate the incidence and mortality rates we observe for known tumor extension and tumor size. Isocitrate dehydrogenase 1/2 mutation and 1p/19q codeletion were used for the classification of gliomas in the 2016 World Health Organization classification of tumors of the central nervous system (46). The SEER database did not record the information of these two molecules before 2018, so we did not include them in the analysis. Glioma incidence and mortality in the larger population need to be monitored to see if this trend is sustained.

## Conclusion

During 1975-2018, the incidence of glioma remained stable on the whole, but a significant decline in incidence was found from 1987 to 2018. Epidemiological changes in non-glioblastoma astrocytoma contributed the most to overall trends in glioma incidence and mortality. Trends in glioma incidence and mortality vary significantly by tumor characteristics. These findings can improve understanding of risk factors and guide the focus of glioma treatment.

## Data Availability Statement

The original contributions presented in the study are included in the article/**Supplementary Material**. Further inquiries can be directed to the corresponding author.

## Author Contributions

The conception and design of the work, DL, MW, and SW. The acquisition, analysis, and interpretation of data, YC, JG, LC, XS, FL, ZC, and TX. Drafting the manuscript, DL, MW, and YC. Revising the manuscript, HS and SW. All authors contributed to the article and approved the submitted version.

## Funding

This study was funded by Key Research and Development Project of Zhejiang Provincial Department of Science and Technology (Grant No. 2021C03105) and Natural Science Foundation of Zhejiang Province (Grant No. Y21H090041).

## Conflict of Interest

The authors declare that the research was conducted in the absence of any commercial or financial relationships that could be construed as a potential conflict of interest.

## Publisher’s Note

All claims expressed in this article are solely those of the authors and do not necessarily represent those of their affiliated organizations, or those of the publisher, the editors and the reviewers. Any product that may be evaluated in this article, or claim that may be made by its manufacturer, is not guaranteed or endorsed by the publisher.
