# Supplementary material for: Trends in Intracranial Glioma Incidence and Mortality in the United States, 1975-2018
Source: Front Oncol. 2021 Nov 1;11:748061. doi: 10.3389/fonc.2021.748061 (PMC8591029; doi:10.3389/fonc.2021.748061)
Supplement: Supplementary file 1 [file DataSheet_1.docx]

Supplementary Data

eTable 1. Definitions of Glioma Histology Groups

eTable 2. WHO Grade of Glioma Histology Codes

eTable 3. Glioma Incidence Rates and Incidence-Based Mortality Rates Grouped by Sex and Age

eTable 4. Glioma Relative Survival (Percent) According to Year of Diagnosis

eTable 5. Glioma Incidence Rates for Known and Unknown Values of Histologic Type, Tumor Extension and Tumor Size

eTable 6. Glioma Incidence-Based Mortality Rates for Known and Unknown Values of Histologic Type, Tumor Extension and Tumor Size

eTable 7. Glioma Incidence Rates During 1975-2018, Overall and According to Sex and Race

eTable 8. Glioma Incidence Rates During 1975-2018 According to Age at Diagnosis

eTable 9. Glioma Incidence Ratesa During 1990-2018 According to Median Household Income and Rural-Urban Distribution

eTable 10. Glioma Incidence Rates During 1975-2018 According to Histologic Type

eTable 11. Glioma Incidence Rates During 1975-2018 According to WHO Grade

eTable 12. Glioblastoma Incidence Rates During 1983-2015 According to Tumor Extension

eTable 13. Glioblastoma Incidence Rates During 1983-2018 According to Tumor Size

eTable 14. Glioma Incidence-Based Mortality Rates During 1995-2018, Overall and According to Sex and Race

eTable 15. Glioma Incidence-Based Mortality Rates During 1995-2018 According to Age at Death

eTable 16. Glioma Incidence-Based Mortality Ratesa During 1995-2018 According to Median Household Income and Rural-Urban Distribution

eTable 17. Glioma Incidence-Based Mortality Rates During 1995-2018 According to Histology Type

eTable 18. Glioma Incidence-Based Mortality Rates During 1995-2018 According to WHO Grade

eTable 19. Glioblastoma Incidence-Based Mortality Rates During 1995-2015 Among Glioblastoma Cases Diagnosed During 1983-2015 According to Tumor Extension

eTable 20. Glioblastoma Incidence-Based Mortality Rates During 1995-2018 Among Glioblastoma Cases Diagnosed During 1983-2018 According to Tumor Size

eTable 1. Definitions of Glioma Histology Groups

| Histology group | CBTRUS histology | ICD-O-3^a^ topography codes | ICD-O-3 Histology codes |
| --- | --- | --- | --- |
| Glioblastoma | Glioblastoma | C71.0-C71.9 | 9440, 9441, 9442 |
| Non-glioblastoma astrocytoma | Pilocytic astrocytoma  Diffuse astrocytoma  Anaplastic astrocytoma  Unique astrocytoma variants | C71.0-C71.9 | 9381, 9384, 9400, 9401, 9410, 9411, 9420, 9421, 9424 |
| Oligodendroglial tumors | Oligodendroglioma  Anaplastic oligodendroglioma  Oligoastrocytic tumors | C71.0-C71.9 | 9382, 9450, 9451, 9460 |
| Ependymoma | Ependymal tumors | C71.0-C71.9 | 9391, 9392, 9393 |
| Glioma, NOS | Glioma malignant, NOS | C71.0-C71.9 | 9380 |

^a^ International Classification of Diseases for Oncology 3^rd^ Edition, 2000. World Health Organization, Geneva, Switzerland.

Abbreviations: CBTRUS, Central Brain Tumor Registry of the United States; NOS, Not otherwise specified.

eTable 2. WHO Grade of Glioma Histology Codes

| WHO classification | ICD-O-3^a^ Histology codes |
| --- | --- |
| Grade I | NA |
| Grade II | 9382, 9391, 9393, 9400, 9410, 9411, 9420, 9421, 9424, 9450 |
| Grade III | 9392, 9401, 9451 |
| Grade IV | 9440, 9441, 9442 |
| NOS | 9380, 9381, 9460 |

^a^ International Classification of Diseases for Oncology 3^rd^ Edition, 2000. World Health Organization, Geneva, Switzerland.

Abbreviations: WHO, World Health Organization; NOS, Not otherwise specified.

eTable 3. Glioma Incidence Rates^a^ and Incidence-Based Mortality Rates^a^ Grouped by Sex and Age

|  | Incidence | | | | | Incidence-based mortality | | | | |
| --- | --- | --- | --- | --- | --- | --- | --- | --- | --- | --- |
| Glioma | Male | | Female | |  | Male | | Female | |  |
| Age group  (year) | No. of Cases | Rate | No. of Cases | Rate | Rate Ratio M/F^b^ (95% CI) | No. of Cases | Rate | No. of Cases | Rate | Rate Ratio M/F^b^ (95% CI) |
| <20 | 3234 | 1.97 | 2865 | 1.83 | 1.08 (1.02-1.13) | 498 | 0.53 | 428 | 0.48 | 1.11 (0.98-1.27) |
| 20-39 | 5189 | 3.08 | 3846 | 2.28 | 1.35 (1.29-1.41) | 1399 | 1.48 | 885 | 0.94 | 1.57 (1.44-1.71) |
| 40-59 | 10799 | 7.68 | 7364 | 5.05 | 1.52 (1.48-1.57) | 5864 | 6.24 | 3778 | 3.89 | 1.61 (1.54-1.67) |
| 60-79 | 13445 | 20.15 | 10542 | 13.00 | 1.55 (1.51-1.59) | 8566 | 20.58 | 6423 | 13.03 | 1.58 (1.53-1.63) |
| ≥80 | 2329 | 19.50 | 2546 | 11.68 | 1.67 (1.58-1.77) | 1995 | 23.85 | 2086 | 14.32 | 1.67 (1.57-1.77) |
|  | | | | | | | | | | |
| Glioblastoma | Male | | Female | |  | Male | | Female | |  |
| Age group  (year) | No. of Cases | Rate | No. of Cases | Rate | Rate Ratio M/F^b^ (95% CI) | No. of Cases | Rate | No. of Cases | Rate | Rate Ratio M/F^b^ (95% CI) |
| <20 | 251 | 0.15 | 183 | 0.12 | 1.31 (1.08-1.59) | 106 | 0.11 | 82 | 0.09 | 1.23 (0.92-1.66) |
| 20-39 | 1104 | 0.68 | 689 | 0.42 | 1.62 (1.47-1.78) | 453 | 0.48 | 271 | 0.29 | 1.66 (1.43-1.94) |
| 40-59 | 6252 | 4.38 | 3979 | 2.66 | 1.65 (1.58-1.71) | 3680 | 3.86 | 2288 | 2.32 | 1.67 (1.58-1.76) |
| 60-79 | 9682 | 14.56 | 7380 | 9.12 | 1.60 (1.55-1.65) | 6533 | 15.75 | 4803 | 9.76 | 1.61 (1.55-1.68) |
| ≥80 | 1720 | 14.38 | 1746 | 8.02 | 1.79 (1.68-1.92) | 1509 | 18.02 | 1476 | 10.18 | 1.77 (1.65-1.90) |

^a^ Rates were calculated as number of cases per 100,000 person-years and age-adjusted to the 2000 U.S. standard population.

^b^ M/F: Male/Female

eTable 4. Glioma Relative Survival^a^ (Percent) According to Year of Diagnosis

| Glioma relative survival (percent) according to year of diagnosis | | | | | | | | | | | | | | | | | | | | | | | |
| --- | --- | --- | --- | --- | --- | --- | --- | --- | --- | --- | --- | --- | --- | --- | --- | --- | --- | --- | --- | --- | --- | --- | --- |
| Survival time | 1975-1979 | 1980-1984 | 1985-1989 | 1990-1994 | 1995-1999 | 2000 | 2001 | 2002 | 2003 | 2004 | 2005 | 2006 | 2007 | 2008 | 2009 | 2010 | 2011 | 2012 | 2013 | 2014 | 2015 | 2016 | 2017 |
| 1-year | 39.24% | 41.75% | 44.55% | 45.88% | 45.56% | 48.53% | 49.66% | 53.20% | 51.39% | 51.68% | 52.68% | 56.02% | 56.46% | 57.67% | 57.65% | 57.23% | 56.39% | 59.50% | 60.30% | 59.96% | 58.00% | 54.39% | 58.40% |
| 2-year | 23.46% | 26.93% | 29.29% | 30.13% | 30.21% | 32.91% | 31.83% | 37.89% | 35.17% | 35.66% | 37.77% | 39.45% | 39.54% | 39.06% | 38.97% | 38.92% | 38.26% | 40.17% | 42.06% | 39.11% | 39.04% | 34.83% |  |
| 3-year | 19.21% | 22.57% | 25.06% | 25.93% | 25.99% | 29.16% | 27.83% | 31.65% | 29.25% | 30.23% | 31.17% | 32.08% | 32.06% | 32.65% | 32.75% | 30.69% | 31.36% | 31.42% | 32.73% | 31.04% | 30.48% |  |  |
| 4-year | 16.75% | 19.73% | 22.26% | 23.64% | 23.70% | 26.65% | 25.23% | 29.19% | 26.85% | 27.61% | 27.77% | 29.50% | 28.10% | 28.92% | 28.62% | 27.29% | 27.32% | 27.77% | 29.68% | 27.93% |  |  |  |
| 5-year | 14.84% | 17.66% | 20.20% | 22.08% | 22.00% | 25.22% | 22.96% | 26.71% | 24.97% | 26.52% | 25.15% | 27.02% | 25.88% | 27.23% | 26.49% | 24.25% | 24.56% | 25.35% | 27.25% |  |  |  |  |
| 6-year | 13.35% | 16.16% | 18.68% | 21.03% | 20.51% | 23.59% | 21.86% | 24.85% | 23.47% | 25.08% | 23.01% | 24.86% | 23.79% | 25.42% | 24.71% | 21.90% | 23.55% | 24.02% |  |  |  |  |  |
| 7-year | 12.40% | 14.91% | 17.72% | 19.61% | 19.63% | 22.05% | 20.95% | 23.75% | 22.31% | 23.61% | 21.95% | 23.90% | 22.67% | 23.90% | 23.71% | 20.73% | 22.36% |  |  |  |  |  |  |
| 8-year | 11.42% | 13.96% | 16.46% | 18.78% | 18.73% | 21.29% | 20.01% | 23.14% | 21.20% | 22.55% | 20.63% | 22.48% | 21.68% | 22.95% | 22.86% | 19.71% |  |  |  |  |  |  |  |
| 9-year | 10.40% | 12.93% | 15.59% | 17.87% | 17.81% | 20.15% | 19.41% | 22.14% | 20.36% | 21.64% | 19.89% | 21.19% | 20.61% | 21.54% | 22.42% |  |  |  |  |  |  |  |  |
| 10-year | 9.73% | 11.84% | 14.86% | 17.19% | 16.95% | 19.18% | 18.99% | 21.38% | 19.76% | 20.73% | 19.06% | 20.50% | 19.66% | 20.59% |  |  |  |  |  |  |  |  |  |
| Glioblastoma relative survival (percent) according to year of diagnosis | | | | | | | | | | | | | | | | | | | | | | | |
| Survival time | 1975-1979 | 1980-1984 | 1985-1989 | 1990-1994 | 1995-1999 | 2000 | 2001 | 2002 | 2003 | 2004 | 2005 | 2006 | 2007 | 2008 | 2009 | 2010 | 2011 | 2012 | 2013 | 2014 | 2015 | 2016 | 2017 |
| 1-year | 26.18% | 24.07% | 28.03% | 27.52% | 26.60% | 28.10% | 31.66% | 33.37% | 33.98% | 35.68% | 36.77% | 38.30% | 41.74% | 43.58% | 42.55% | 44.55% | 43.24% | 46.38% | 47.39% | 47.27% | 43.38% | 42.35% | 44.90% |
| 2-year | 7.99% | 7.72% | 8.42% | 7.90% | 7.39% | 8.69% | 8.54% | 11.61% | 13.23% | 14.30% | 18.18% | 16.87% | 19.28% | 18.35% | 19.07% | 20.90% | 20.45% | 21.86% | 23.20% | 19.61% | 18.71% | 17.79% |  |
| 3-year | 4.98% | 4.75% | 4.80% | 4.57% | 3.94% | 5.03% | 5.09% | 5.85% | 6.68% | 8.65% | 11.09% | 9.40% | 10.79% | 11.17% | 12.72% | 11.87% | 12.24% | 11.11% | 12.13% | 10.78% | 9.63% |  |  |
| 4-year | 3.99% | 3.44% | 3.21% | 3.23% | 2.83% | 3.95% | 3.36% | 5.13% | 5.16% | 6.36% | 8.99% | 6.97% | 7.40% | 7.08% | 8.50% | 7.84% | 7.71% | 7.41% | 8.07% | 7.68% |  |  |  |
| 5-year | 3.25% | 2.95% | 2.77% | 2.89% | 2.39% | 3.64% | 2.87% | 4.11% | 3.89% | 5.57% | 6.86% | 5.63% | 5.51% | 6.06% | 6.11% | 5.55% | 5.27% | 6.02% | 5.89% |  |  |  |  |
| 6-year | 2.71% | 2.44% | 2.48% | 2.66% | 2.08% | 3.00% | 2.37% | 3.67% | 2.42% | 4.35% | 5.37% | 4.74% | 4.42% | 4.74% | 4.98% | 4.33% | 4.65% | 4.94% |  |  |  |  |  |
| 7-year | 2.36% | 2.24% | 2.23% | 2.32% | 1.86% | 2.67% | 2.03% | 3.36% | 2.29% | 3.51% | 4.44% | 4.13% | 3.61% | 3.67% | 4.61% | 3.51% | 4.02% |  |  |  |  |  |  |
| 8-year | 2.06% | 2.00% | 1.93% | 2.19% | 1.76% | 2.36% | 1.66% | 3.06% | 2.01% | 2.79% | 4.06% | 3.21% | 3.50% | 3.28% | 4.37% | 3.14% |  |  |  |  |  |  |  |
| 9-year | 1.65% | 1.64% | 1.80% | 2.06% | 1.64% | 2.19% | 1.66% | 2.44% | 1.57% | 2.66% | 3.79% | 2.27% | 2.96% | 2.59% | 4.26% |  |  |  |  |  |  |  |  |
| 10-year | 1.54% | 1.53% | 1.71% | 1.89% | 1.36% | 1.70% | 1.66% | 2.12% | 1.26% | 2.39% | 3.69% | 2.13% | 2.84% | 2.33% |  |  |  |  |  |  |  |  |  |

^a^Cases less than 15 years of age will be excluded from the analysis.

eTable 5. Glioma Incidence Rates^a^ for Known and Unknown Values of Histologic Type, Tumor Extension and Tumor Size

| Glioma incidence by histologic type | | | | | | | | | | | | |
| --- | --- | --- | --- | --- | --- | --- | --- | --- | --- | --- | --- | --- |
|  | Total | | Glioblastoma | | Non-glioblastoma astrocytoma | | Oligodendroglial tumors | | Ependymoma | | Glioma, NOS | |
| Year of diagnosis | No. of Cases | Rate | No. of Cases | Rate | No. of Cases | Rate | No. of Cases | Rate | No. of Cases | Rate | No. of Cases | Rate |
| 1975-79 (R1) | 4799 | 5.05 | 2229 | 2.41 | 1828 | 1.87 | 210 | 0.22 | 113 | 0.11 | 419 | 0.44 |
| 1980-84 | 5535 | 5.49 | 2218 | 2.25 | 2372 | 2.31 | 342 | 0.34 | 115 | 0.10 | 488 | 0.49 |
| 1985-89 | 6484 | 6.03 | 2786 | 2.67 | 2605 | 2.36 | 420 | 0.38 | 152 | 0.13 | 521 | 0.49 |
| 1990-94 | 6810 | 5.89 | 3379 | 3.02 | 2226 | 1.87 | 631 | 0.51 | 149 | 0.12 | 425 | 0.37 |
| 1995-99 | 7110 | 5.72 | 3753 | 3.09 | 1745 | 1.38 | 980 | 0.75 | 168 | 0.13 | 464 | 0.37 |
| 2000-04 | 7608 | 5.72 | 4128 | 3.14 | 1696 | 1.26 | 1021 | 0.75 | 196 | 0.15 | 567 | 0.43 |
| 2005-09 | 8013 | 5.60 | 4646 | 3.22 | 1662 | 1.18 | 907 | 0.64 | 211 | 0.15 | 587 | 0.42 |
| 2010-2014 | 8617 | 5.53 | 5203 | 3.23 | 1831 | 1.24 | 805 | 0.53 | 201 | 0.14 | 577 | 0.39 |
| 2015-18 (R2) | 7183 | 5.31 | 4551 | 3.20 | 1441 | 1.16 | 472 | 0.38 | 169 | 0.14 | 550 | 0.44 |
| Rate Ratio (R2/R1) 95% CI |  | 1.05 (1.01-1.09) |  | 1.33 (1.26-1.40) |  | 0.62 (0.57-0.66) |  | 1.69 (1.43-2.01) |  | 1.33 (1.04-1.72) |  | 1.00 (0.88-1.14) |
| Glioblastoma incidence by tumor extension^b^ | | | | | | | | | | | | |
|  | Localized | | Regional | | Distant | | Unknown | |  |  |  |  |
| Year of diagnosis | No. of Cases | Rate | No. of Cases | Rate | No. of Cases | Rate | No. of Cases | Rate |  |  |  |  |
| 1983-87 (R1) | 1913 | 1.87 | 362 | 0.35 | 17 | 0.02 | 249 | 0.25 |  |  |  |  |
| 1988-92 | 2301 | 2.12 | 501 | 0.46 | 28 | 0.03 | 309 | 0.29 |  |  |  |  |
| 1993-97 | 2623 | 2.23 | 631 | 0.53 | 19 | 0.02 | 321 | 0.28 |  |  |  |  |
| 1998-02 | 2911 | 2.29 | 756 | 0.59 | 21 | 0.02 | 246 | 0.19 |  |  |  |  |
| 2003-07 | 3475 | 2.51 | 839 | 0.61 | - | - | 176 | 0.13 |  |  |  |  |
| 2008-11 | 3119 | 2.56 | 699 | 0.57 | - | - | 131 | 0.11 |  |  |  |  |
| 2012-15 (R2) | 3332 | 2.50 | 835 | 0.63 | - | - | 175 | 0.13 |  |  |  |  |
| Rate Ratio (R2/R1) 95% CI |  | 1.34 (1.26-1.42) |  | 1.79 (1.58-2.03) |  | - |  | 0.54 (0.44-0.66) |  |  |  |  |
| Glioblastoma incidence by tumor size^c^ | | | | | | | | | | | | |
|  | ≤3.0 | | >3.0 to ≤5.0 | | >5.0 | | Unknown | |  |  |  |  |
| Year of diagnosis | No. of Cases | Rate | No. of Cases | Rate | No. of Cases | Rate | No. of Cases | Rate |  |  |  |  |
| 1983-87 (R1) | 218 | 0.22 | 326 | 0.32 | 226 | 0.22 | 1771 | 1.72 |  |  |  |  |
| 1988-92 | 384 | 0.36 | 646 | 0.60 | 336 | 0.31 | 1773 | 1.64 |  |  |  |  |
| 1993-97 | 496 | 0.42 | 917 | 0.78 | 557 | 0.47 | 1624 | 1.38 |  |  |  |  |
| 1998-02 | 563 | 0.44 | 1204 | 0.95 | 759 | 0.59 | 1408 | 1.11 |  |  |  |  |
| 2003-07 | 744 | 0.54 | 1511 | 1.08 | 1198 | 0.87 | 1051 | 0.76 |  |  |  |  |
| 2008-12 | 894 | 0.58 | 1793 | 1.17 | 1439 | 0.93 | 892 | 0.58 |  |  |  |  |
| 2013-18 (R2) | 1285 | 0.61 | 2416 | 1.15 | 2046 | 0.98 | 969 | 0.47 |  |  |  |  |
| Rate Ratio (R2/R1) 95% CI |  | 2.86 (2.47-3.33) |  | 3.57 (3.17-4.03) |  | 4.41 (3.84-5.10) |  | 0.28 (0.25-0.30) |  |  |  |  |

-Statistic suppressed due to <16 cases in the time interval.

^a^ Rates were calculated as number of cases per 100,000 person-years and age-adjusted to the 2000 U.S. standard population.

^b^ Tumor extension was based on cases diagnosed between 1983 and 2015.

^c^ Tumor size was based on cases diagnosed between 1983 and 2018.

Abbreviations: NOS, Not otherwise specified.

eTable 6. Glioma Incidence-Based Mortality Rates^a^ for Known and Unknown Values of Histologic Type, Tumor Extension and Tumor Size

| Glioma incidence-based mortality by histologic type | | | | | | | | | | | | |
| --- | --- | --- | --- | --- | --- | --- | --- | --- | --- | --- | --- | --- |
|  | Total | | Glioblastoma | | Non-glioblastoma astrocytoma | | Oligodendroglial tumors | | Ependymoma | | Glioma, NOS | |
| Year of death | No. of Cases | Rate | No. of Cases | Rate | No. of Cases | Rate | No. of Cases | Rate | No. of Cases | Rate | No. of Cases | Rate |
| 1995-98 (R1) | 4650 | 4.80 | 2890 | 3.01 | 1111 | 1.13 | 299 | 0.30 | 66 | 0.06 | 284 | 0.29 |
| 1999-02 | 4933 | 4.78 | 3070 | 3.00 | 984 | 0.94 | 487 | 0.46 | 68 | 0.06 | 324 | 0.31 |
| 2003-06 | 5030 | 4.60 | 3241 | 2.98 | 918 | 0.83 | 468 | 0.42 | 74 | 0.07 | 329 | 0.30 |
| 2007-10 | 5303 | 4.49 | 3564 | 3.00 | 876 | 0.74 | 470 | 0.40 | 70 | 0.06 | 323 | 0.28 |
| 2011-14 | 5747 | 4.47 | 3935 | 3.03 | 927 | 0.73 | 476 | 0.38 | 90 | 0.07 | 319 | 0.26 |
| 2015-18 (R2) | 6259 | 4.50 | 4423 | 3.11 | 987 | 0.75 | 441 | 0.33 | 85 | 0.06 | 323 | 0.25 |
| Rate Ratio (R2/R1) 95% CI |  | 0.94 (0.90-0.97) |  | 1.03 (0.98-1.08) |  | 0.66 (0.61-0.72) |  | 1.09 (0.94-1.28) |  | 1.01 (0.72-1.42) |  | 0.84 (0.71-0.99) |
| Glioblastoma incidence-based mortality by tumor extension^b^ | | | | | | | | | | | | |
|  | Localized | | Regional | | Distant | | Unknown | |  |  |  |  |
| Year of death | No. of Cases | Rate | No. of Cases | Rate | No. of Cases | Rate | No. of Cases | Rate |  |  |  |  |
| 1995-98 (R1) | 2113 | 2.20 | 501 | 0.52 | 16 | 0.02 | 253 | 0.27 |  |  |  |  |
| 1999-02 | 2262 | 2.21 | 593 | 0.58 | 20 | 0.02 | 193 | 0.19 |  |  |  |  |
| 2003-06 | 2469 | 2.27 | 629 | 0.57 | - | - | 143 | 0.13 |  |  |  |  |
| 2007-10 | 2760 | 2.32 | 683 | 0.57 | - | - | 126 | 0.11 |  |  |  |  |
| 2011-15 (R2) | 3894 | 2.37 | 943 | 0.57 | - | - | 203 | 0.13 |  |  |  |  |
| Rate Ratio (R2/R1) 95% CI |  | 1.08 (1.02-1.14) |  | 1.10 (0.98-1.23) |  | - |  | 0.47 (0.39-0.57) |  |  |  |  |
| Glioblastoma incidence-based mortality by tumor size^c^ | | | | | | | | | | | | |
|  | ≤3.0 | | >3.0 to ≤5.0 | | >5.0 | | Unknown | |  |  |  |  |
| Year of death | No. of Cases | Rate | No. of Cases | Rate | No. of Cases | Rate | No. of Cases | Rate |  |  |  |  |
| 1995-98 (R1) | 394 | 0.41 | 746 | 0.78 | 475 | 0.49 | 1268 | 1.32 |  |  |  |  |
| 1999-02 | 439 | 0.43 | 933 | 0.91 | 582 | 0.57 | 1114 | 1.09 |  |  |  |  |
| 2003-06 | 523 | 0.49 | 1057 | 0.97 | 790 | 0.72 | 886 | 0.81 |  |  |  |  |
| 2007-10 | 601 | 0.51 | 1242 | 1.04 | 1044 | 0.88 | 684 | 0.58 |  |  |  |  |
| 2011-14 | 715 | 0.55 | 1409 | 1.09 | 1143 | 0.87 | 688 | 0.54 |  |  |  |  |
| 2015-18 (R2) | 823 | 0.58 | 1632 | 1.15 | 1339 | 0.94 | 650 | 0.47 |  |  |  |  |
| Rate Ratio (R2/R1) 95% CI |  | 1.40 (1.24-1.59) |  | 1.47 (1.35-1.61) |  | 1.91 (1.72-2.12) |  | 0.35 (0.32-0.39) |  |  |  |  |

-Statistic suppressed due to <16 cases in the time interval.

^a^Rates were calculated as number of cases per 100,000 person-years and age-adjusted to the 2000 U.S. standard population. Based on cases diagnosed between 1975 and 2018.

^b^Tumor extension was based on cases diagnosed between 1983 and 2015.

^c^Tumor size was based on cases diagnosed between 1983 and 2018.

Abbreviations: NOS, Not otherwise specified.

eTable 7. Glioma Incidence Rates^a^ During 1975-2018, Overall and According to Sex and Race

| Year of diagnosis | Overall | | Sex | | | | Race | | | | | |
| --- | --- | --- | --- | --- | --- | --- | --- | --- | --- | --- | --- | --- |
|  |  |  | Male | | Female | | White | | Black | | Other^b^ | |
|  | No. cases | Rate | No. cases | Rate | No. cases | Rate | No. cases | Rate | No. cases | Rate | No. cases | Rate |
| 1975 | 875 | 4.69 | 493 | 5.64 | 382 | 3.88 | 813 | 5.00 | 46 | 2.91 | 16 | 1.53 |
| 1976 | 909 | 4.89 | 533 | 6.17 | 376 | 3.79 | 847 | 5.22 | 37 | 2.76 | 25 | 2.99 |
| 1977 | 1009 | 5.30 | 575 | 6.62 | 434 | 4.24 | 940 | 5.72 | 44 | 2.75 | 25 | 2.11 |
| 1978 | 978 | 5.05 | 535 | 6.03 | 443 | 4.25 | 905 | 5.40 | 52 | 3.16 | 21 | 2.11 |
| 1979 | 1028 | 5.32 | 585 | 6.48 | 443 | 4.32 | 950 | 5.72 | 57 | 3.47 | 21 | 1.82 |
| 1980 | 1059 | 5.37 | 591 | 6.49 | 468 | 4.44 | 986 | 5.84 | 47 | 2.46 | 26 | 2.08 |
| 1981 | 1123 | 5.65 | 645 | 7.04 | 478 | 4.49 | 1023 | 6.03 | 74 | 4.21 | 26 | 1.95 |
| 1982 | 1096 | 5.46 | 627 | 6.85 | 469 | 4.37 | 1013 | 5.93 | 53 | 3.11 | 30 | 2.17 |
| 1983 | 1147 | 5.60 | 642 | 6.83 | 505 | 4.57 | 1075 | 6.16 | 38 | 2.26 | 34 | 2.76 |
| 1984 | 1110 | 5.33 | 612 | 6.38 | 498 | 4.43 | 1013 | 5.73 | 62 | 3.62 | 35 | 2.51 |
| 1985 | 1265 | 6.03 | 717 | 7.48 | 548 | 4.86 | 1150 | 6.47 | 74 | 4.21 | 41 | 3.14 |
| 1986 | 1272 | 5.99 | 691 | 7.02 | 581 | 5.12 | 1155 | 6.47 | 74 | 3.51 | 43 | 2.78 |
| 1987 | 1340 | 6.19 | 727 | 7.28 | 613 | 5.27 | 1231 | 6.79 | 68 | 3.39 | 41 | 2.39 |
| 1988 | 1300 | 5.98 | 710 | 7.04 | 590 | 5.08 | 1184 | 6.52 | 74 | 3.74 | 42 | 2.53 |
| 1989 | 1307 | 5.97 | 746 | 7.43 | 561 | 4.72 | 1182 | 6.50 | 84 | 4.05 | 41 | 2.22 |
| 1990 | 1354 | 6.04 | 728 | 7.00 | 626 | 5.26 | 1221 | 6.58 | 75 | 3.28 | 58 | 3.08 |
| 1991 | 1358 | 6.07 | 770 | 7.57 | 588 | 4.85 | 1247 | 6.73 | 57 | 2.59 | 54 | 2.85 |
| 1992 | 1383 | 5.97 | 802 | 7.58 | 581 | 4.67 | 1259 | 6.59 | 66 | 3.03 | 58 | 2.90 |
| 1993 | 1356 | 5.76 | 750 | 6.90 | 606 | 4.80 | 1224 | 6.33 | 71 | 3.13 | 61 | 3.05 |
| 1994 | 1359 | 5.65 | 760 | 6.90 | 599 | 4.62 | 1215 | 6.19 | 82 | 3.28 | 62 | 2.81 |
| 1995 | 1353 | 5.59 | 758 | 6.79 | 595 | 4.62 | 1204 | 6.12 | 79 | 3.42 | 70 | 3.12 |
| 1996 | 1388 | 5.69 | 802 | 7.15 | 586 | 4.48 | 1241 | 6.27 | 75 | 3.16 | 72 | 3.16 |
| 1997 | 1422 | 5.72 | 796 | 6.90 | 626 | 4.72 | 1278 | 6.36 | 75 | 3.08 | 69 | 2.87 |
| 1998 | 1426 | 5.66 | 776 | 6.68 | 650 | 4.81 | 1272 | 6.26 | 77 | 2.97 | 77 | 3.12 |
| 1999 | 1521 | 5.95 | 880 | 7.46 | 641 | 4.68 | 1349 | 6.57 | 89 | 3.38 | 83 | 3.09 |
| 2000 | 1494 | 5.75 | 845 | 7.06 | 649 | 4.70 | 1333 | 6.44 | 90 | 3.36 | 71 | 2.60 |
| 2001 | 1473 | 5.61 | 827 | 6.81 | 646 | 4.63 | 1310 | 6.29 | 82 | 3.10 | 81 | 2.91 |
| 2002 | 1539 | 5.79 | 861 | 6.98 | 678 | 4.83 | 1363 | 6.49 | 89 | 3.24 | 87 | 3.08 |
| 2003 | 1530 | 5.68 | 838 | 6.67 | 692 | 4.87 | 1337 | 6.30 | 98 | 3.49 | 95 | 3.25 |
| 2004 | 1572 | 5.78 | 878 | 6.84 | 694 | 4.81 | 1393 | 6.51 | 86 | 3.04 | 93 | 3.00 |
| 2005 | 1584 | 5.74 | 857 | 6.64 | 727 | 4.95 | 1395 | 6.44 | 99 | 3.54 | 90 | 2.86 |
| 2006 | 1529 | 5.44 | 875 | 6.65 | 654 | 4.41 | 1343 | 6.11 | 99 | 3.32 | 87 | 2.73 |
| 2007 | 1581 | 5.55 | 888 | 6.61 | 693 | 4.63 | 1373 | 6.20 | 119 | 3.98 | 89 | 2.64 |
| 2008 | 1632 | 5.63 | 920 | 6.79 | 712 | 4.68 | 1444 | 6.45 | 87 | 2.78 | 101 | 2.86 |
| 2009 | 1687 | 5.67 | 947 | 6.75 | 740 | 4.74 | 1467 | 6.39 | 125 | 3.84 | 95 | 2.62 |
| 2010 | 1653 | 5.50 | 915 | 6.46 | 738 | 4.68 | 1440 | 6.20 | 111 | 3.44 | 102 | 2.81 |
| 2011 | 1729 | 5.63 | 979 | 6.79 | 750 | 4.64 | 1506 | 6.41 | 114 | 3.50 | 109 | 2.87 |
| 2012 | 1743 | 5.57 | 984 | 6.69 | 759 | 4.59 | 1501 | 6.29 | 121 | 3.43 | 121 | 2.97 |
| 2013 | 1757 | 5.59 | 983 | 6.57 | 774 | 4.71 | 1512 | 6.32 | 120 | 3.42 | 125 | 3.11 |
| 2014 | 1735 | 5.37 | 1010 | 6.56 | 725 | 4.32 | 1510 | 6.16 | 107 | 2.85 | 118 | 2.77 |
| 2015 | 1839 | 5.60 | 1063 | 6.81 | 776 | 4.56 | 1593 | 6.42 | 113 | 3.03 | 133 | 3.06 |
| 2016 | 1766 | 5.23 | 1014 | 6.32 | 752 | 4.29 | 1524 | 5.98 | 113 | 3.05 | 129 | 2.83 |
| 2017 | 1786 | 5.25 | 1026 | 6.30 | 760 | 4.37 | 1547 | 6.03 | 118 | 3.12 | 121 | 2.71 |
| 2018 | 1792 | 5.19 | 1035 | 6.34 | 757 | 4.16 | 1556 | 6.01 | 125 | 3.09 | 111 | 2.42 |

^a^ Rates were calculated as number of cases per 100,000 person-years and age-adjusted to the 2000 U.S. standard population.

^b^ Includes American Indian/Alaskan Native and Asian/Pacific Islander.

eTable 8. Glioma Incidence Rates^a^ During 1975-2018 According to Age at Diagnosis

| Year of diagnosis | Age at Diagnosis | | | | | | | | | |
| --- | --- | --- | --- | --- | --- | --- | --- | --- | --- | --- |
|  | <20 | | 20-39 | | 40-59 | | 60-79 | | ≥80 | |
|  | No. cases | Rate | No. cases | Rate | No. cases | Rate | No. cases | Rate | No. cases | Rate |
| 1975 | 93 | 1.29 | 129 | 2.34 | 304 | 6.54 | 330 | 13.44 | 19 | 4.55 |
| 1976 | 85 | 1.21 | 136 | 2.35 | 293 | 6.37 | 374 | 15.35 | 21 | 4.87 |
| 1977 | 138 | 1.97 | 144 | 2.34 | 338 | 7.29 | 353 | 14.17 | 36 | 8.14 |
| 1978 | 120 | 1.75 | 140 | 2.21 | 306 | 6.49 | 374 | 14.63 | 38 | 8.26 |
| 1979 | 105 | 1.57 | 194 | 3.10 | 325 | 7.00 | 368 | 14.29 | 36 | 7.62 |
| 1980 | 111 | 1.64 | 155 | 2.37 | 345 | 7.19 | 405 | 15.35 | 43 | 8.90 |
| 1981 | 106 | 1.59 | 175 | 2.50 | 341 | 7.53 | 453 | 16.41 | 48 | 9.76 |
| 1982 | 90 | 1.35 | 179 | 2.49 | 331 | 7.22 | 446 | 16.10 | 50 | 9.98 |
| 1983 | 102 | 1.53 | 180 | 2.52 | 328 | 6.98 | 484 | 17.12 | 53 | 10.25 |
| 1984 | 114 | 1.74 | 207 | 2.80 | 299 | 6.37 | 433 | 15.13 | 57 | 10.76 |
| 1985 | 123 | 1.86 | 218 | 2.91 | 337 | 7.17 | 513 | 17.60 | 74 | 13.60 |
| 1986 | 147 | 2.21 | 226 | 2.97 | 329 | 7.05 | 502 | 16.99 | 68 | 12.18 |
| 1987 | 140 | 2.10 | 236 | 3.07 | 342 | 7.11 | 550 | 18.30 | 72 | 12.51 |
| 1988 | 136 | 2.04 | 224 | 2.84 | 345 | 7.17 | 533 | 17.79 | 62 | 10.43 |
| 1989 | 151 | 2.23 | 186 | 2.37 | 353 | 7.08 | 529 | 17.48 | 88 | 14.47 |
| 1990 | 146 | 2.12 | 228 | 2.86 | 381 | 7.49 | 522 | 16.89 | 77 | 12.26 |
| 1991 | 145 | 2.09 | 215 | 2.70 | 393 | 7.67 | 503 | 16.25 | 102 | 15.89 |
| 1992 | 154 | 2.22 | 223 | 2.79 | 365 | 6.73 | 546 | 17.39 | 95 | 14.15 |
| 1993 | 143 | 1.99 | 223 | 2.77 | 338 | 6.09 | 565 | 17.96 | 87 | 12.53 |
| 1994 | 137 | 1.90 | 251 | 3.06 | 357 | 6.16 | 518 | 16.39 | 96 | 13.43 |
| 1995 | 146 | 1.99 | 197 | 2.42 | 396 | 6.59 | 521 | 16.44 | 93 | 12.59 |
| 1996 | 149 | 2.03 | 187 | 2.31 | 421 | 6.83 | 499 | 15.72 | 132 | 17.23 |
| 1997 | 114 | 1.54 | 222 | 2.75 | 429 | 6.68 | 558 | 17.56 | 99 | 12.59 |
| 1998 | 127 | 1.68 | 207 | 2.56 | 402 | 6.02 | 560 | 17.56 | 130 | 16.13 |
| 1999 | 138 | 1.82 | 247 | 3.08 | 424 | 6.14 | 572 | 17.90 | 140 | 16.94 |
| 2000 | 160 | 2.11 | 224 | 2.82 | 477 | 6.71 | 496 | 15.43 | 137 | 16.17 |
| 2001 | 176 | 2.31 | 207 | 2.62 | 450 | 6.11 | 511 | 15.87 | 129 | 14.82 |
| 2002 | 169 | 2.22 | 237 | 2.98 | 450 | 5.94 | 556 | 17.14 | 127 | 14.13 |
| 2003 | 152 | 1.99 | 221 | 2.81 | 485 | 6.28 | 547 | 16.68 | 125 | 13.65 |
| 2004 | 138 | 1.81 | 219 | 2.85 | 508 | 6.46 | 559 | 16.78 | 148 | 15.80 |
| 2005 | 152 | 1.99 | 216 | 2.80 | 490 | 6.08 | 557 | 16.48 | 169 | 17.68 |
| 2006 | 129 | 1.67 | 205 | 2.63 | 494 | 6.05 | 545 | 15.79 | 156 | 16.05 |
| 2007 | 151 | 1.95 | 196 | 2.53 | 512 | 6.17 | 578 | 16.34 | 144 | 14.59 |
| 2008 | 164 | 2.11 | 234 | 3.01 | 514 | 6.16 | 555 | 15.10 | 165 | 16.48 |
| 2009 | 175 | 2.24 | 225 | 2.89 | 496 | 5.78 | 581 | 15.05 | 210 | 20.74 |
| 2010 | 184 | 2.35 | 187 | 2.38 | 473 | 5.54 | 634 | 16.04 | 175 | 17.09 |
| 2011 | 144 | 1.85 | 231 | 2.94 | 502 | 5.77 | 686 | 16.70 | 166 | 16.14 |
| 2012 | 147 | 1.89 | 227 | 2.87 | 498 | 5.72 | 688 | 16.02 | 183 | 17.49 |
| 2013 | 165 | 2.12 | 221 | 2.70 | 527 | 6.09 | 687 | 15.93 | 157 | 15.06 |
| 2014 | 159 | 2.05 | 211 | 2.59 | 525 | 5.98 | 673 | 14.69 | 167 | 15.79 |
| 2015 | 163 | 2.09 | 221 | 2.71 | 508 | 5.77 | 759 | 16.09 | 188 | 17.36 |
| 2016 | 113 | 1.46 | 223 | 2.65 | 455 | 5.24 | 799 | 16.24 | 176 | 16.15 |
| 2017 | 141 | 1.82 | 205 | 2.42 | 493 | 5.76 | 784 | 15.31 | 163 | 14.95 |
| 2018 | 157 | 2.02 | 196 | 2.29 | 484 | 5.60 | 781 | 14.82 | 174 | 15.68 |

^a^ Rates were calculated as number of cases per 100,000 person-years and age-adjusted to the 2000 U.S. standard population.

eTable 9. Glioma Incidence Rates^a^ During 1990-2018 According to Median Household Income and Rural-Urban Distribution

| Year of diagnosis | Median Household Income | | | | Rural-Urban Distribution | | | |
| --- | --- | --- | --- | --- | --- | --- | --- | --- |
|  | <75000 | | ≥75000 | | Urban | | Rural | |
|  | No. cases | Rate | No. cases | Rate | No. cases | Rate | No. cases | Rate |
| 1990 | 747 | 5.99 | 606 | 6.11 | 1105 | 6.12 | 204 | 6.27 |
| 1991 | 770 | 6.22 | 588 | 5.86 | 1126 | 6.25 | 204 | 6.19 |
| 1992 | 770 | 5.97 | 610 | 5.94 | 1131 | 6.05 | 210 | 6.06 |
| 1993 | 724 | 5.49 | 631 | 6.08 | 1121 | 5.92 | 194 | 5.55 |
| 1994 | 766 | 5.70 | 592 | 5.59 | 1125 | 5.80 | 196 | 5.64 |
| 1995 | 753 | 5.56 | 598 | 5.60 | 1085 | 5.56 | 225 | 6.36 |
| 1996 | 637 | 5.56 | 751 | 5.80 | 1140 | 5.81 | 195 | 5.47 |
| 1997 | 681 | 5.88 | 740 | 5.59 | 1168 | 5.84 | 203 | 5.70 |
| 1998 | 661 | 5.65 | 764 | 5.68 | 1215 | 5.82 | 173 | 5.53 |
| 1999 | 696 | 5.91 | 825 | 6.00 | 1291 | 6.08 | 183 | 5.87 |
| 2000 | 692 | 5.69 | 802 | 5.84 | 1305 | 5.80 | 189 | 5.50 |
| 2001 | 701 | 5.70 | 771 | 5.53 | 1268 | 5.58 | 204 | 5.81 |
| 2002 | 743 | 5.96 | 796 | 5.62 | 1329 | 5.76 | 210 | 6.02 |
| 2003 | 682 | 5.41 | 847 | 5.94 | 1333 | 5.70 | 196 | 5.58 |
| 2004 | 756 | 5.93 | 815 | 5.65 | 1367 | 5.78 | 204 | 5.67 |
| 2005 | 763 | 5.89 | 821 | 5.62 | 1380 | 5.76 | 204 | 5.60 |
| 2006 | 884 | 5.44 | 644 | 5.44 | 1319 | 5.41 | 209 | 5.58 |
| 2007 | 936 | 5.67 | 645 | 5.39 | 1364 | 5.51 | 217 | 5.73 |
| 2008 | 952 | 5.68 | 680 | 5.56 | 1436 | 5.64 | 196 | 5.66 |
| 2009 | 1023 | 5.69 | 664 | 5.62 | 1466 | 5.58 | 221 | 6.36 |
| 2010 | 1034 | 5.30 | 619 | 5.89 | 1470 | 5.56 | 183 | 5.15 |
| 2011 | 1161 | 5.75 | 568 | 5.42 | 1520 | 5.62 | 209 | 5.71 |
| 2012 | 1209 | 5.69 | 534 | 5.29 | 1504 | 5.44 | 239 | 6.60 |
| 2013 | 1144 | 5.57 | 613 | 5.64 | 1565 | 5.63 | 192 | 5.15 |
| 2014 | 1142 | 5.41 | 593 | 5.28 | 1524 | 5.32 | 211 | 5.82 |
| 2015 | 1044 | 5.49 | 795 | 5.77 | 1668 | 5.75 | 171 | 4.42 |
| 2016 | 1013 | 5.23 | 753 | 5.25 | 1563 | 5.24 | 203 | 5.22 |
| 2017 | 992 | 5.42 | 794 | 5.04 | 1572 | 5.22 | 214 | 5.50 |
| 2018 | 943 | 5.07 | 848 | 5.33 | 1583 | 5.17 | 208 | 5.42 |

^a^ Rates were calculated as number of cases per 100,000 person-years and age-adjusted to the 2000 U.S. standard population.

eTable 10. Glioma Incidence Rates^a^ During 1975-2018 According to Histologic Type

| Year of diagnosis | Histologic Type | | | | | | | | | |
| --- | --- | --- | --- | --- | --- | --- | --- | --- | --- | --- |
|  | Glioblastoma | | Non-glioblastoma astrocytoma | | Oligodendroglial tumors | | Ependymoma | | Glioma, NOS | |
|  | No. cases | Rate | No. cases | Rate | No. cases | Rate | No. cases | Rate | No. cases | Rate |
| 1975 | 540 | 2.95 | 201 | 1.04 | 34 | 0.18 | 17 | 0.08 | 83 | 0.44 |
| 1976 | 499 | 2.75 | 269 | 1.39 | 44 | 0.24 | 28 | 0.14 | 69 | 0.37 |
| 1977 | 401 | 2.19 | 457 | 2.34 | 51 | 0.26 | 24 | 0.11 | 76 | 0.39 |
| 1978 | 378 | 1.99 | 429 | 2.19 | 44 | 0.23 | 24 | 0.11 | 103 | 0.53 |
| 1979 | 411 | 2.20 | 472 | 2.37 | 37 | 0.19 | 20 | 0.10 | 88 | 0.47 |
| 1980 | 430 | 2.24 | 465 | 2.32 | 57 | 0.28 | 16 | 0.07 | 91 | 0.46 |
| 1981 | 438 | 2.27 | 480 | 2.38 | 70 | 0.34 | 28 | 0.13 | 107 | 0.54 |
| 1982 | 440 | 2.24 | 473 | 2.30 | 71 | 0.36 | 25 | 0.11 | 87 | 0.44 |
| 1983 | 490 | 2.45 | 461 | 2.22 | 67 | 0.32 | 25 | 0.11 | 104 | 0.51 |
| 1984 | 420 | 2.06 | 493 | 2.31 | 77 | 0.38 | 21 | 0.10 | 99 | 0.49 |
| 1985 | 558 | 2.74 | 499 | 2.30 | 79 | 0.36 | 25 | 0.11 | 104 | 0.51 |
| 1986 | 553 | 2.67 | 533 | 2.48 | 68 | 0.30 | 27 | 0.12 | 91 | 0.42 |
| 1987 | 520 | 2.47 | 613 | 2.76 | 84 | 0.37 | 25 | 0.11 | 98 | 0.47 |
| 1988 | 551 | 2.62 | 494 | 2.20 | 94 | 0.43 | 39 | 0.16 | 122 | 0.58 |
| 1989 | 604 | 2.85 | 466 | 2.07 | 95 | 0.41 | 36 | 0.15 | 106 | 0.49 |
| 1990 | 627 | 2.89 | 489 | 2.12 | 110 | 0.46 | 38 | 0.16 | 90 | 0.41 |
| 1991 | 676 | 3.12 | 441 | 1.92 | 124 | 0.52 | 32 | 0.13 | 85 | 0.39 |
| 1992 | 680 | 3.02 | 459 | 1.92 | 124 | 0.51 | 33 | 0.13 | 87 | 0.39 |
| 1993 | 686 | 2.99 | 439 | 1.81 | 122 | 0.50 | 23 | 0.09 | 86 | 0.36 |
| 1994 | 710 | 3.05 | 398 | 1.62 | 151 | 0.58 | 23 | 0.09 | 77 | 0.32 |
| 1995 | 679 | 2.88 | 401 | 1.63 | 158 | 0.61 | 35 | 0.13 | 80 | 0.33 |
| 1996 | 747 | 3.14 | 323 | 1.29 | 181 | 0.71 | 34 | 0.13 | 103 | 0.42 |
| 1997 | 770 | 3.17 | 338 | 1.34 | 187 | 0.73 | 34 | 0.13 | 93 | 0.37 |
| 1998 | 733 | 2.98 | 341 | 1.33 | 235 | 0.89 | 30 | 0.11 | 87 | 0.34 |
| 1999 | 824 | 3.29 | 342 | 1.31 | 219 | 0.83 | 35 | 0.13 | 101 | 0.39 |
| 2000 | 776 | 3.04 | 366 | 1.39 | 194 | 0.72 | 42 | 0.16 | 116 | 0.45 |
| 2001 | 789 | 3.06 | 320 | 1.20 | 218 | 0.80 | 36 | 0.13 | 110 | 0.42 |
| 2002 | 807 | 3.07 | 358 | 1.33 | 220 | 0.81 | 39 | 0.15 | 115 | 0.43 |
| 2003 | 854 | 3.21 | 314 | 1.16 | 197 | 0.71 | 41 | 0.15 | 124 | 0.46 |
| 2004 | 902 | 3.33 | 338 | 1.23 | 192 | 0.70 | 38 | 0.14 | 102 | 0.38 |
| 2005 | 939 | 3.40 | 323 | 1.18 | 182 | 0.65 | 35 | 0.13 | 105 | 0.38 |
| 2006 | 850 | 3.01 | 315 | 1.14 | 207 | 0.74 | 45 | 0.16 | 112 | 0.40 |
| 2007 | 935 | 3.26 | 324 | 1.14 | 161 | 0.58 | 44 | 0.16 | 117 | 0.42 |
| 2008 | 946 | 3.23 | 354 | 1.24 | 167 | 0.58 | 39 | 0.14 | 126 | 0.45 |
| 2009 | 976 | 3.20 | 346 | 1.21 | 190 | 0.65 | 48 | 0.16 | 127 | 0.45 |
| 2010 | 969 | 3.15 | 371 | 1.27 | 168 | 0.57 | 43 | 0.15 | 102 | 0.36 |
| 2011 | 1049 | 3.34 | 363 | 1.23 | 163 | 0.55 | 39 | 0.13 | 115 | 0.39 |
| 2012 | 1056 | 3.25 | 358 | 1.21 | 172 | 0.57 | 43 | 0.15 | 114 | 0.39 |
| 2013 | 1056 | 3.25 | 369 | 1.23 | 170 | 0.56 | 35 | 0.12 | 127 | 0.43 |
| 2014 | 1073 | 3.16 | 370 | 1.26 | 132 | 0.43 | 41 | 0.13 | 119 | 0.39 |
| 2015 | 1131 | 3.29 | 376 | 1.23 | 139 | 0.44 | 51 | 0.17 | 142 | 0.47 |
| 2016 | 1166 | 3.31 | 333 | 1.06 | 108 | 0.35 | 35 | 0.12 | 124 | 0.39 |
| 2017 | 1124 | 3.12 | 366 | 1.17 | 115 | 0.37 | 41 | 0.14 | 140 | 0.45 |
| 2018 | 1130 | 3.09 | 366 | 1.16 | 110 | 0.35 | 42 | 0.14 | 144 | 0.45 |

^a^ Rates were calculated as number of cases per 100,000 person-years and age-adjusted to the 2000 U.S. standard population.

Abbreviations: NOS, Not otherwise specified.

eTable 11. Glioma Incidence Rates^a^ During 1975-2018 According to WHO Grade

| Year of diagnosis | WHO Grade | | | | | | | | | |
| --- | --- | --- | --- | --- | --- | --- | --- | --- | --- | --- |
|  | Grade I | | Grade II | | Grade III | | Grade IV | | Unknown | |
|  | No. cases | Rate | No. cases | Rate | No. cases | Rate | No. cases | Rate | No. cases | Rate |
| 1975 | - | - | 245 | 1.26 | 5 | 0.03 | 540 | 2.95 | 85 | 0.46 |
| 1976 | - | - | 320 | 1.66 | 19 | 0.10 | 499 | 2.75 | 71 | 0.38 |
| 1977 | - | - | 488 | 2.48 | 44 | 0.24 | 401 | 2.19 | 76 | 0.39 |
| 1978 | - | - | 468 | 2.39 | 29 | 0.14 | 378 | 1.99 | 103 | 0.53 |
| 1979 | - | - | 500 | 2.50 | 28 | 0.14 | 411 | 2.20 | 89 | 0.48 |
| 1980 | - | - | 514 | 2.55 | 22 | 0.10 | 430 | 2.24 | 93 | 0.48 |
| 1981 | - | - | 535 | 2.64 | 42 | 0.20 | 438 | 2.27 | 108 | 0.54 |
| 1982 | - | - | 532 | 2.61 | 37 | 0.17 | 440 | 2.24 | 87 | 0.44 |
| 1983 | - | - | 494 | 2.36 | 56 | 0.27 | 490 | 2.45 | 107 | 0.53 |
| 1984 | - | - | 554 | 2.61 | 36 | 0.17 | 420 | 2.06 | 100 | 0.49 |
| 1985 | - | - | 545 | 2.52 | 58 | 0.26 | 558 | 2.74 | 104 | 0.51 |
| 1986 | - | - | 553 | 2.54 | 75 | 0.35 | 553 | 2.67 | 91 | 0.42 |
| 1987 | - | - | 620 | 2.78 | 102 | 0.47 | 520 | 2.47 | 98 | 0.47 |
| 1988 | - | - | 531 | 2.36 | 93 | 0.42 | 551 | 2.62 | 125 | 0.59 |
| 1989 | - | - | 482 | 2.14 | 114 | 0.49 | 604 | 2.85 | 107 | 0.49 |
| 1990 | - | - | 515 | 2.21 | 120 | 0.52 | 627 | 2.89 | 92 | 0.42 |
| 1991 | - | - | 465 | 1.98 | 129 | 0.57 | 676 | 3.12 | 88 | 0.40 |
| 1992 | - | - | 476 | 1.97 | 137 | 0.58 | 680 | 3.02 | 90 | 0.40 |
| 1993 | - | - | 461 | 1.90 | 121 | 0.49 | 686 | 2.99 | 88 | 0.37 |
| 1994 | - | - | 465 | 1.85 | 105 | 0.43 | 710 | 3.05 | 79 | 0.33 |
| 1995 | - | - | 469 | 1.86 | 122 | 0.50 | 679 | 2.88 | 83 | 0.35 |
| 1996 | - | - | 410 | 1.61 | 125 | 0.51 | 747 | 3.14 | 106 | 0.43 |
| 1997 | - | - | 419 | 1.63 | 137 | 0.55 | 770 | 3.17 | 96 | 0.38 |
| 1998 | - | - | 446 | 1.71 | 153 | 0.59 | 733 | 2.98 | 94 | 0.37 |
| 1999 | - | - | 444 | 1.68 | 148 | 0.57 | 824 | 3.29 | 105 | 0.40 |
| 2000 | - | - | 443 | 1.66 | 157 | 0.59 | 776 | 3.04 | 118 | 0.46 |
| 2001 | - | - | 426 | 1.58 | 143 | 0.53 | 789 | 3.06 | 115 | 0.44 |
| 2002 | - | - | 457 | 1.68 | 158 | 0.59 | 807 | 3.07 | 117 | 0.44 |
| 2003 | - | - | 416 | 1.52 | 130 | 0.47 | 854 | 3.21 | 130 | 0.48 |
| 2004 | - | - | 446 | 1.63 | 117 | 0.43 | 902 | 3.33 | 107 | 0.39 |
| 2005 | - | - | 404 | 1.46 | 135 | 0.49 | 939 | 3.40 | 106 | 0.38 |
| 2006 | - | - | 408 | 1.47 | 159 | 0.57 | 850 | 3.01 | 112 | 0.40 |
| 2007 | - | - | 383 | 1.37 | 137 | 0.48 | 935 | 3.26 | 126 | 0.45 |
| 2008 | - | - | 420 | 1.48 | 136 | 0.47 | 946 | 3.23 | 130 | 0.46 |
| 2009 | - | - | 430 | 1.48 | 149 | 0.52 | 976 | 3.20 | 132 | 0.47 |
| 2010 | - | - | 409 | 1.40 | 166 | 0.57 | 969 | 3.15 | 109 | 0.38 |
| 2011 | - | - | 409 | 1.38 | 149 | 0.50 | 1049 | 3.34 | 121 | 0.41 |
| 2012 | - | - | 384 | 1.30 | 182 | 0.60 | 1056 | 3.25 | 121 | 0.40 |
| 2013 | - | - | 375 | 1.26 | 196 | 0.63 | 1056 | 3.25 | 130 | 0.44 |
| 2014 | - | - | 343 | 1.16 | 191 | 0.63 | 1073 | 3.16 | 126 | 0.41 |
| 2015 | - | - | 331 | 1.10 | 228 | 0.72 | 1131 | 3.29 | 149 | 0.49 |
| 2016 | - | - | 267 | 0.88 | 207 | 0.65 | 1166 | 3.31 | 126 | 0.40 |
| 2017 | - | - | 280 | 0.92 | 240 | 0.75 | 1124 | 3.12 | 142 | 0.46 |
| 2018 | - | - | 310 | 1.01 | 205 | 0.63 | 1130 | 3.09 | 147 | 0.46 |

-Statistic suppressed due to <16 cases in the time interval.

^a^ Rates were calculated as number of cases per 100,000 person-years and age-adjusted to the 2000 U.S. standard population.

Abbreviations: WHO, World Health Organization.

eTable 12. Glioblastoma Incidence Rates^a^ During 1983-2015 According to Tumor Extension^b^

| Year of diagnosis | Tumor extension | | | | | | | |
| --- | --- | --- | --- | --- | --- | --- | --- | --- |
|  | Localized | | Regional | | Distant | | Unknown | |
|  | No. cases | Rate | No. cases | Rate | No. cases | Rate | No. cases | Rate |
| 1983 | 375 | 1.88 | 71 | 0.35 | - | - | 43 | 0.22 |
| 1984 | 330 | 1.64 | 58 | 0.27 | - | - | 32 | 0.16 |
| 1985 | 424 | 2.07 | 71 | 0.36 | - | - | 58 | 0.29 |
| 1986 | 403 | 1.95 | 77 | 0.36 | - | - | 69 | 0.35 |
| 1987 | 381 | 1.79 | 85 | 0.42 | - | - | 47 | 0.23 |
| 1988 | 428 | 2.02 | 71 | 0.34 | - | - | 50 | 0.25 |
| 1989 | 435 | 2.05 | 108 | 0.51 | - | - | 50 | 0.24 |
| 1990 | 458 | 2.12 | 106 | 0.48 | - | - | 56 | 0.26 |
| 1991 | 483 | 2.21 | 116 | 0.55 | - | - | 72 | 0.34 |
| 1992 | 497 | 2.22 | 100 | 0.44 | - | - | 81 | 0.36 |
| 1993 | 476 | 2.08 | 143 | 0.62 | - | - | 66 | 0.29 |
| 1994 | 522 | 2.24 | 123 | 0.52 | - | - | 60 | 0.27 |
| 1995 | 511 | 2.17 | 95 | 0.40 | - | - | 69 | 0.30 |
| 1996 | 549 | 2.30 | 127 | 0.53 | - | - | 66 | 0.28 |
| 1997 | 565 | 2.32 | 143 | 0.58 | - | - | 60 | 0.25 |
| 1998 | 524 | 2.13 | 150 | 0.61 | - | - | 55 | 0.23 |
| 1999 | 611 | 2.44 | 148 | 0.59 | - | - | 62 | 0.25 |
| 2000 | 574 | 2.25 | 157 | 0.62 | - | - | 38 | 0.15 |
| 2001 | 586 | 2.27 | 154 | 0.60 | - | - | 47 | 0.19 |
| 2002 | 616 | 2.35 | 147 | 0.56 | - | - | 44 | 0.17 |
| 2003 | 658 | 2.48 | 150 | 0.56 | - | - | 39 | 0.15 |
| 2004 | 705 | 2.61 | 156 | 0.57 | - | - | 44 | 0.16 |
| 2005 | 728 | 2.63 | 186 | 0.68 | - | - | 30 | 0.11 |
| 2006 | 663 | 2.35 | 162 | 0.56 | - | - | 30 | 0.11 |
| 2007 | 721 | 2.51 | 185 | 0.66 | - | - | 33 | 0.12 |
| 2008 | 748 | 2.55 | 171 | 0.58 | - | - | 29 | 0.10 |
| 2009 | 790 | 2.59 | 161 | 0.53 | - | - | 27 | 0.09 |
| 2010 | 739 | 2.41 | 194 | 0.63 | - | - | 38 | 0.12 |
| 2011 | 842 | 2.70 | 173 | 0.54 | - | - | 37 | 0.12 |
| 2012 | 835 | 2.58 | 186 | 0.57 | - | - | 45 | 0.13 |
| 2013 | 812 | 2.49 | 210 | 0.65 | - | - | 39 | 0.12 |
| 2014 | 816 | 2.39 | 224 | 0.66 | - | - | 37 | 0.12 |
| 2015 | 869 | 2.54 | 215 | 0.61 | - | - | 54 | 0.16 |

-Statistic suppressed due to <16 cases in the time interval

^a^ Rates were calculated as number of cases per 100,000 person-years and age-adjusted to the 2000 U.S. standard population.

^b^ Based on cases diagnosed between 1983 and 2015.

eTable 13. Glioblastoma Incidence Rates^a^ During 1983-2018 According to Tumor Size^b^

| Year of diagnosis | Tumor size | | | | | | | |
| --- | --- | --- | --- | --- | --- | --- | --- | --- |
|  | ≤3.0 | | >3.0 to ≤5.0 | | >5.0 | | Unknown | |
|  | No. cases | Rate | No. cases | Rate | No. cases | Rate | No. cases | Rate |
| 1983 | 40 | 0.20 | 48 | 0.24 | 37 | 0.18 | 365 | 1.82 |
| 1984 | 27 | 0.12 | 54 | 0.27 | 43 | 0.22 | 296 | 1.45 |
| 1985 | 56 | 0.28 | 55 | 0.27 | 54 | 0.26 | 393 | 1.93 |
| 1986 | 47 | 0.23 | 80 | 0.39 | 45 | 0.22 | 381 | 1.83 |
| 1987 | 48 | 0.23 | 89 | 0.43 | 47 | 0.23 | 336 | 1.59 |
| 1988 | 59 | 0.28 | 103 | 0.49 | 59 | 0.27 | 336 | 1.58 |
| 1989 | 75 | 0.36 | 109 | 0.51 | 68 | 0.33 | 352 | 1.66 |
| 1990 | 75 | 0.36 | 133 | 0.61 | 64 | 0.30 | 355 | 1.63 |
| 1991 | 69 | 0.32 | 165 | 0.76 | 79 | 0.37 | 363 | 1.68 |
| 1992 | 106 | 0.47 | 136 | 0.62 | 66 | 0.29 | 373 | 1.65 |
| 1993 | 87 | 0.38 | 183 | 0.80 | 92 | 0.40 | 324 | 1.42 |
| 1994 | 89 | 0.38 | 172 | 0.74 | 102 | 0.43 | 347 | 1.50 |
| 1995 | 100 | 0.43 | 176 | 0.75 | 99 | 0.42 | 304 | 1.29 |
| 1996 | 108 | 0.46 | 188 | 0.78 | 125 | 0.52 | 328 | 1.38 |
| 1997 | 112 | 0.46 | 198 | 0.81 | 139 | 0.57 | 321 | 1.33 |
| 1998 | 103 | 0.42 | 218 | 0.89 | 131 | 0.53 | 281 | 1.15 |
| 1999 | 113 | 0.46 | 240 | 0.96 | 141 | 0.56 | 331 | 1.32 |
| 2000 | 105 | 0.41 | 242 | 0.95 | 174 | 0.68 | 255 | 1.00 |
| 2001 | 117 | 0.46 | 252 | 0.97 | 147 | 0.57 | 275 | 1.08 |
| 2002 | 125 | 0.48 | 252 | 0.96 | 166 | 0.63 | 266 | 1.01 |
| 2003 | 117 | 0.45 | 282 | 1.05 | 178 | 0.66 | 280 | 1.06 |
| 2004 | 156 | 0.58 | 286 | 1.05 | 229 | 0.85 | 236 | 0.87 |
| 2005 | 182 | 0.66 | 309 | 1.11 | 254 | 0.92 | 201 | 0.73 |
| 2006 | 129 | 0.46 | 335 | 1.19 | 245 | 0.86 | 146 | 0.52 |
| 2007 | 160 | 0.57 | 299 | 1.02 | 292 | 1.02 | 188 | 0.66 |
| 2008 | 153 | 0.52 | 344 | 1.18 | 276 | 0.95 | 175 | 0.59 |
| 2009 | 194 | 0.64 | 356 | 1.17 | 281 | 0.92 | 148 | 0.49 |
| 2010 | 170 | 0.55 | 318 | 1.03 | 282 | 0.91 | 203 | 0.67 |
| 2011 | 186 | 0.60 | 401 | 1.26 | 280 | 0.90 | 185 | 0.60 |
| 2012 | 191 | 0.59 | 374 | 1.17 | 320 | 0.96 | 181 | 0.57 |
| 2013 | 200 | 0.62 | 368 | 1.12 | 336 | 1.03 | 158 | 0.50 |
| 2014 | 206 | 0.59 | 374 | 1.11 | 336 | 0.99 | 162 | 0.48 |
| 2015 | 194 | 0.57 | 432 | 1.22 | 329 | 0.98 | 183 | 0.55 |
| 2016 | 224 | 0.63 | 428 | 1.23 | 365 | 1.03 | 156 | 0.45 |
| 2017 | 227 | 0.64 | 417 | 1.16 | 346 | 0.95 | 136 | 0.38 |
| 2018 | 234 | 0.64 | 397 | 1.06 | 334 | 0.93 | 174 | 0.48 |

^a^ Rates were calculated as number of cases per 100,000 person-years and age-adjusted to the 2000 U.S. standard population.

^b^ Based on cases diagnosed between 1983 and 2016.

eTable 14. Glioma Incidence-Based Mortality Rates^a,b^ During 1995-2018, Overall and According to Sex and Race

| Year of death | Overall | | Sex | | | | Race | | | | | |
| --- | --- | --- | --- | --- | --- | --- | --- | --- | --- | --- | --- | --- |
|  |  |  | Male | | Female | | White | | Black | | Other^c^ | |
|  | No. cases | Rate | No. cases | Rate | No. cases | Rate | No. cases | Rate | No. cases | Rate | No. cases | Rate |
| 1995 | 1115 | 4.70 | 618 | 5.80 | 497 | 3.81 | 1008 | 5.15 | 53 | 2.55 | 54 | 2.57 |
| 1996 | 1178 | 4.91 | 667 | 6.20 | 511 | 3.89 | 1065 | 5.39 | 57 | 2.76 | 56 | 2.63 |
| 1997 | 1161 | 4.77 | 643 | 5.77 | 518 | 3.90 | 1041 | 5.21 | 62 | 2.86 | 58 | 2.56 |
| 1998 | 1196 | 4.83 | 663 | 5.92 | 533 | 3.95 | 1097 | 5.41 | 46 | 1.98 | 53 | 2.26 |
| 1999 | 1240 | 4.93 | 71768 | 6.32 | 523 | 3.81 | 1122 | 5.48 | 63 | 2.77 | 55 | 2.23 |
| 2000 | 1210 | 4.73 | 706 | 6.06 | 504 | 3.62 | 1081 | 5.21 | 83 | 3.24 | 46 | 1.79 |
| 2001 | 1222 | 4.70 | 708 | 6.00 | 514 | 3.64 | 1099 | 5.23 | 62 | 2.60 | 61 | 2.38 |
| 2002 | 1261 | 4.78 | 751 | 6.23 | 510 | 3.59 | 1122 | 5.30 | 65 | 2.61 | 74 | 2.67 |
| 2003 | 1221 | 4.60 | 690 | 5.68 | 531 | 3.69 | 1097 | 5.17 | 68 | 2.82 | 56 | 2.04 |
| 2004 | 1229 | 4.52 | 672 | 5.38 | 557 | 3.79 | 1078 | 4.96 | 78 | 2.86 | 73 | 2.47 |
| 2005 | 1348 | 4.88 | 738 | 5.83 | 610 | 4.09 | 1217 | 5.54 | 66 | 2.48 | 65 | 2.20 |
| 2006 | 1232 | 4.38 | 701 | 5.42 | 531 | 3.53 | 1094 | 4.89 | 72 | 2.65 | 66 | 2.09 |
| 2007 | 1286 | 4.51 | 737 | 5.57 | 549 | 3.59 | 1154 | 5.13 | 73 | 2.64 | 59 | 1.78 |
| 2008 | 1318 | 4.52 | 789 | 5.92 | 529 | 3.36 | 1172 | 5.11 | 76 | 2.72 | 70 | 2.11 |
| 2009 | 1298 | 4.33 | 737 | 5.35 | 561 | 3.47 | 1155 | 4.91 | 79 | 2.58 | 64 | 1.87 |
| 2010 | 1401 | 4.60 | 801 | 5.67 | 600 | 3.71 | 1231 | 5.16 | 98 | 3.21 | 72 | 1.96 |
| 2011 | 1379 | 4.44 | 773 | 5.39 | 606 | 3.61 | 1212 | 4.98 | 86 | 2.72 | 81 | 2.22 |
| 2012 | 1433 | 4.48 | 845 | 5.74 | 588 | 3.42 | 1256 | 5.04 | 93 | 3.00 | 84 | 2.14 |
| 2013 | 1450 | 4.51 | 793 | 5.29 | 657 | 3.83 | 1278 | 5.15 | 71 | 2.06 | 101 | 2.49 |
| 2014 | 1485 | 4.47 | 850 | 5.51 | 635 | 3.57 | 1290 | 5.03 | 109 | 2.96 | 86 | 2.06 |
| 2015 | 1561 | 4.68 | 920 | 5.92 | 641 | 3.59 | 1369 | 5.36 | 95 | 2.66 | 97 | 2.26 |
| 2016 | 1584 | 4.56 | 968 | 6.02 | 616 | 3.31 | 1378 | 5.17 | 99 | 2.72 | 107 | 2.40 |
| 2017 | 1557 | 4.42 | 928 | 5.59 | 629 | 3.43 | 1344 | 4.99 | 96 | 2.66 | 117 | 2.51 |
| 2018 | 1557 | 4.34 | 907 | 5.44 | 650 | 4.43 | 1337 | 4.90 | 108 | 2.72 | 112 | 2.39 |

^a^ Rates were calculated as number of deaths per 100,000 person-years and age-adjusted to the 2000 U.S. standard population.

^b^ Based on cases diagnosed between 1975 and 2016.

^c^ Includes American Indian/Alaskan Native and Asian/Pacific Islander.

eTable 15. Glioma Incidence-Based Mortality Rates^a,b^ During 1995-2018 According to Age at Death

| Year of death | Age at Death | | | | | | | | | |
| --- | --- | --- | --- | --- | --- | --- | --- | --- | --- | --- |
|  | <20 | | 20-39 | | 40-59 | | 60-79 | | ≥80 | |
|  | No. cases | Rate | No. cases | Rate | No. cases | Rate | No. cases | Rate | No. cases | Rate |
| 1995 | 29 | 0.40 | 110 | 1.36 | 349 | 5.88 | 526 | 16.59 | 101 | 13.67 |
| 1996 | 40 | 0.55 | 82 | 1.01 | 371 | 6.02 | 547 | 17.24 | 138 | 18.05 |
| 1997 | 34 | 0.46 | 104 | 1.31 | 372 | 5.87 | 539 | 16.94 | 112 | 14.23 |
| 1998 | 34 | 0.46 | 110 | 1.38 | 347 | 5.25 | 571 | 17.92 | 134 | 16.65 |
| 1999 | 37 | 0.48 | 103 | 1.30 | 363 | 5.29 | 580 | 18.15 | 157 | 19.00 |
| 2000 | 39 | 0.51 | 100 | 1.27 | 392 | 5.51 | 532 | 16.57 | 147 | 17.39 |
| 2001 | 37 | 0.49 | 103 | 1.31 | 402 | 5.45 | 535 | 16.60 | 145 | 16.66 |
| 2002 | 44 | 0.59 | 109 | 1.39 | 398 | 5.20 | 572 | 17.61 | 138 | 15.39 |
| 2003 | 46 | 0.61 | 110 | 1.44 | 382 | 4.97 | 550 | 16.80 | 133 | 14.58 |
| 2004 | 38 | 0.51 | 86 | 1.12 | 409 | 5.14 | 540 | 16.24 | 156 | 16.68 |
| 2005 | 46 | 0.61 | 107 | 1.40 | 420 | 5.14 | 596 | 17.66 | 179 | 18.71 |
| 2006 | 37 | 0.49 | 84 | 1.11 | 420 | 5.07 | 521 | 15.17 | 170 | 17.44 |
| 2007 | 33 | 0.43 | 104 | 1.38 | 432 | 5.15 | 558 | 15.88 | 159 | 16.16 |
| 2008 | 33 | 0.43 | 81 | 1.06 | 422 | 5.03 | 607 | 16.56 | 175 | 17.42 |
| 2009 | 49 | 0.63 | 88 | 1.13 | 386 | 4.47 | 574 | 15.10 | 201 | 19.66 |
| 2010 | 49 | 0.63 | 98 | 1.28 | 413 | 4.74 | 630 | 16.01 | 211 | 20.65 |
| 2011 | 43 | 0.56 | 74 | 0.98 | 408 | 4.70 | 664 | 16.24 | 190 | 18.41 |
| 2012 | 41 | 0.53 | 88 | 1.13 | 408 | 4.56 | 687 | 16.21 | 209 | 19.88 |
| 2013 | 28 | 0.37 | 106 | 1.36 | 409 | 4.65 | 732 | 16.92 | 175 | 16.89 |
| 2014 | 46 | 0.60 | 80 | 1.03 | 449 | 5.03 | 713 | 15.55 | 197 | 18.60 |
| 2015 | 34 | 0.44 | 94 | 1.19 | 458 | 5.23 | 745 | 16.03 | 230 | 21.47 |
| 2016 | 34 | 0.44 | 87 | 1.07 | 441 | 4.87 | 825 | 16.97 | 197 | 18.17 |
| 2017 | 34 | 0.44 | 92 | 1.13 | 420 | 4.74 | 809 | 16.04 | 202 | 18.29 |
| 2018 | 41 | 0.53 | 84 | 1.01 | 371 | 4.24 | 836 | 15.98 | 225 | 20.26 |

^a^ Rates were calculated as number of deaths per 100,000 person-years and age-adjusted to the 2000 U.S. standard population.

^b^ Based on cases diagnosed between 1975 and 2016.

eTable 16. Glioma Incidence-Based Mortality Rates^a^ During 1995-2018 According to Median Household Income and Rural-Urban Distribution

| Year of death | Median Household Income | | | | Rural-Urban Distribution | | | |
| --- | --- | --- | --- | --- | --- | --- | --- | --- |
|  | <75000 | | ≥75000 | | Urban | | Rural | |
|  | No. cases | Rate | No. cases | Rate | No. cases | Rate | No. cases | Rate |
| 1995 | 543 | 2.29 | 474 | 2.00 | 800 | 3.38 | 178 | 0.76 |
| 1996 | 565 | 2.35 | 536 | 2.24 | 899 | 3.75 | 165 | 0.70 |
| 1997 | 515 | 2.11 | 561 | 2.32 | 892 | 3.67 | 149 | 0.62 |
| 1998 | 566 | 2.29 | 556 | 2.25 | 921 | 3.72 | 167 | 0.68 |
| 1999 | 560 | 2.23 | 605 | 2.41 | 960 | 3.82 | 172 | 0.69 |
| 2000 | 555 | 2.18 | 603 | 2.36 | 993 | 3.88 | 141 | 0.56 |
| 2001 | 560 | 2.15 | 606 | 2.34 | 1000 | 3.85 | 157 | 0.61 |
| 2002 | 578 | 2.19 | 625 | 2.37 | 1036 | 3.92 | 158 | 0.60 |
| 2003 | 569 | 2.15 | 595 | 2.24 | 963 | 3.63 | 197 | 0.75 |
| 2004 | 555 | 2.05 | 631 | 2.32 | 1018 | 3.74 | 164 | 0.62 |
| 2005 | 630 | 2.28 | 664 | 2.42 | 1104 | 3.99 | 189 | 0.69 |
| 2006 | 621 | 2.22 | 559 | 1.98 | 1029 | 3.66 | 148 | 0.53 |
| 2007 | 690 | 2.43 | 549 | 1.92 | 1064 | 3.73 | 175 | 0.62 |
| 2008 | 744 | 2.55 | 536 | 1.85 | 1102 | 3.78 | 176 | 0.61 |
| 2009 | 752 | 2.53 | 510 | 1.69 | 1066 | 3.56 | 196 | 0.65 |
| 2010 | 791 | 2.61 | 572 | 1.87 | 1186 | 3.88 | 176 | 0.60 |
| 2011 | 848 | 2.72 | 499 | 1.61 | 1191 | 3.83 | 152 | 0.49 |
| 2012 | 894 | 2.80 | 512 | 1.59 | 1222 | 3.81 | 179 | 0.57 |
| 2013 | 923 | 2.87 | 480 | 1.51 | 1227 | 3.82 | 173 | 0.54 |
| 2014 | 936 | 2.81 | 522 | 1.57 | 1270 | 3.82 | 187 | 0.56 |
| 2015 | 939 | 2.80 | 576 | 1.73 | 1325 | 3.98 | 189 | 0.55 |
| 2016 | 949 | 2.71 | 613 | 1.79 | 1380 | 3.98 | 180 | 0.51 |
| 2017 | 871 | 2.49 | 651 | 1.83 | 1339 | 3.81 | 181 | 0.50 |
| 2018 | 848 | 2.35 | 675 | 1.89 | 1341 | 3.74 | 181 | 0.50 |

^a^ Rates were calculated as number of cases per 100,000 person-years and age-adjusted to the 2000 U.S. standard population.

eTable 17. Glioma Incidence-Based Mortality Rates^a,b^ During 1995-2018 According to Histology Type

| Year of death | Histologic Type | | | | | | | | | |
| --- | --- | --- | --- | --- | --- | --- | --- | --- | --- | --- |
|  | Glioblastoma | | Non-glioblastoma astrocytoma | | Oligodendroglial tumors | | Ependymoma | | Glioma, NOS | |
|  | No. cases | Rate | No. cases | Rate | No. cases | Rate | No. cases | Rate | No. cases | Rate |
| 1995 | 701 | 2.98 | 282 | 1.18 | 56 | 0.23 | 16 | 0.06 | 60 | 0.25 |
| 1996 | 738 | 3.10 | 287 | 1.18 | 68 | 0.28 | 13 | 0.05 | 72 | 0.30 |
| 1997 | 712 | 2.95 | 265 | 1.07 | 84 | 0.33 | 20 | 0.08 | 80 | 0.33 |
| 1998 | 739 | 3.01 | 277 | 1.11 | 91 | 0.36 | 17 | 0.07 | 72 | 0.29 |
| 1999 | 767 | 3.08 | 247 | 0.98 | 124 | 0.48 | 16 | 0.06 | 86 | 0.34 |
| 2000 | 763 | 3.01 | 234 | 0.90 | 117 | 0.45 | 17 | 0.07 | 79 | 0.31 |
| 2001 | 748 | 2.91 | 257 | 0.97 | 117 | 0.43 | 15 | 0.06 | 85 | 0.33 |
| 2002 | 792 | 3.02 | 246 | 0.92 | 129 | 0.48 | 20 | 0.07 | 74 | 0.29 |
| 2003 | 791 | 3.00 | 224 | 0.83 | 104 | 0.38 | 18 | 0.07 | 84 | 0.32 |
| 2004 | 780 | 2.90 | 234 | 0.84 | 113 | 0.41 | 15 | 0.06 | 87 | 0.33 |
| 2005 | 889 | 3.24 | 234 | 0.84 | 131 | 0.47 | 20 | 0.08 | 74 | 0.27 |
| 2006 | 781 | 2.77 | 226 | 0.81 | 120 | 0.42 | 21 | 0.08 | 84 | 0.30 |
| 2007 | 851 | 2.98 | 225 | 0.79 | 120 | 0.42 | 23 | 0.08 | 67 | 0.24 |
| 2008 | 878 | 3.00 | 229 | 0.79 | 120 | 0.41 | 16 | 0.06 | 75 | 0.27 |
| 2009 | 894 | 2.98 | 184 | 0.60 | 110 | 0.37 | 20 | 0.07 | 90 | 0.31 |
| 2010 | 941 | 3.05 | 238 | 0.80 | 120 | 0.40 | 11 | 0.04 | 91 | 0.31 |
| 2011 | 925 | 2.95 | 241 | 0.78 | 120 | 0.41 | 22 | 0.07 | 71 | 0.24 |
| 2012 | 979 | 3.02 | 208 | 0.66 | 119 | 0.38 | 30 | 0.10 | 97 | 0.32 |
| 2013 | 991 | 3.07 | 256 | 0.82 | 108 | 0.34 | 13 | 0.04 | 80 | 0.25 |
| 2014 | 1040 | 3.08 | 220 | 0.69 | 129 | 0.40 | 25 | 0.08 | 71 | 0.22 |
| 2015 | 1080 | 3.16 | 265 | 0.85 | 128 | 0.39 | 22 | 0.07 | 66 | 0.21 |
| 2016 | 1152 | 3.26 | 233 | 0.70 | 101 | 0.31 | 19 | 0.06 | 79 | 0.24 |
| 2017 | 1100 | 3.07 | 232 | 0.69 | 125 | 0.37 | 21 | 0.07 | 79 | 0.23 |
| 2018 | 1091 | 2.95 | 257 | 0.77 | 87 | 0.26 | 23 | 0.07 | 99 | 0.30 |

^a^ Rates were calculated as number of deaths per 100,000 person-years and age-adjusted to the 2000 U.S. standard population.

^b^ Based on cases diagnosed between 1975 and 2018.

Abbreviations: NOS, Not otherwise specified.

eTable 18. Glioma Incidence-Based Mortality Rates^a,b^ During 1995-2018 According to WHO Grade

| Year of death | WHO Grade | | | | | | | | | |
| --- | --- | --- | --- | --- | --- | --- | --- | --- | --- | --- |
|  | Grade I | | Grade II | | Grade III | | Grade IV | | Unknown | |
|  | No. cases | Rate | No. cases | Rate | No. cases | Rate | No. cases | Rate | No. cases | Rate |
| 1995 | - | - | 265 | 1.10 | 89 | 0.37 | 701 | 2.98 | 60 | 0.25 |
| 1996 | - | - | 263 | 1.08 | 103 | 0.42 | 738 | 3.10 | 74 | 0.31 |
| 1997 | - | - | 272 | 1.09 | 95 | 0.38 | 712 | 2.95 | 82 | 0.34 |
| 1998 | - | - | 259 | 1.03 | 121 | 0.48 | 739 | 3.01 | 77 | 0.31 |
| 1999 | - | - | 259 | 1.01 | 125 | 0.49 | 767 | 3.08 | 89 | 0.35 |
| 2000 | - | - | 242 | 0.93 | 124 | 0.48 | 763 | 3.01 | 81 | 0.32 |
| 2001 | - | - | 256 | 0.97 | 129 | 0.48 | 748 | 2.91 | 89 | 0.34 |
| 2002 | - | - | 263 | 0.97 | 130 | 0.49 | 792 | 3.02 | 76 | 0.29 |
| 2003 | - | - | 251 | 0.92 | 89 | 0.33 | 791 | 3.00 | 90 | 0.34 |
| 2004 | - | - | 254 | 0.92 | 100 | 0.35 | 780 | 2.90 | 95 | 0.36 |
| 2005 | - | - | 282 | 1.01 | 101 | 0.37 | 889 | 3.24 | 76 | 0.27 |
| 2006 | - | - | 249 | 0.88 | 117 | 0.42 | 781 | 2.77 | 85 | 0.30 |
| 2007 | - | - | 260 | 0.91 | 104 | 0.36 | 851 | 2.98 | 71 | 0.25 |
| 2008 | - | - | 246 | 0.84 | 117 | 0.40 | 878 | 3.00 | 77 | 0.28 |
| 2009 | - | - | 229 | 0.77 | 80 | 0.26 | 894 | 2.98 | 95 | 0.33 |
| 2010 | - | - | 259 | 0.87 | 105 | 0.35 | 941 | 3.05 | 96 | 0.33 |
| 2011 | - | - | 264 | 0.86 | 115 | 0.38 | 925 | 2.95 | 75 | 0.25 |
| 2012 | - | - | 240 | 0.76 | 110 | 0.36 | 979 | 3.02 | 104 | 0.35 |
| 2013 | - | - | 267 | 0.84 | 108 | 0.34 | 991 | 3.07 | 84 | 0.26 |
| 2014 | - | - | 225 | 0.71 | 144 | 0.44 | 1040 | 3.08 | 75 | 0.23 |
| 2015 | - | - | 272 | 0.87 | 139 | 0.43 | 1080 | 3.16 | 70 | 0.22 |
| 2016 | - | - | 209 | 0.63 | 136 | 0.42 | 1152 | 3.26 | 87 | 0.27 |
| 2017 |  |  | 221 | 0.66 | 151 | 0.44 | 1100 | 3.07 | 85 | 0.25 |
| 2018 |  |  | 209 | 0.63 | 153 | 0.45 | 1091 | 2.95 | 104 | 0.32 |

-Statistic suppressed due to <16 cases in the time interval

^a^ Rates were calculated as number of deaths per 100,000 person-years and age-adjusted to the 2000 U.S. standard population.

^b^ Based on cases diagnosed between 1975 and 2018.

Abbreviations: WHO, World Health Organization

eTable 19. Glioblastoma Incidence-Based Mortality Rates^a^ During 1995-2015 Among Glioblastoma Cases Diagnosed During 1983-2015 According to Tumor Extension

| Year of death | Tumor extension | | | | | | | |
| --- | --- | --- | --- | --- | --- | --- | --- | --- |
|  | Localized | | Regional | | Distant | | Unknown | |
|  | No. cases | Rate | No. cases | Rate | No. cases | Rate | No. cases | Rate |
| 1995 | 515 | 2.18 | 107 | 0.46 | - | - | 70 | 0.31 |
| 1996 | 553 | 2.33 | 117 | 0.49 | - | - | 61 | 0.26 |
| 1997 | 528 | 2.19 | 129 | 0.54 | - | - | 52 | 0.22 |
| 1998 | 517 | 2.11 | 148 | 0.60 | - | - | 70 | 0.29 |
| 1999 | 554 | 2.23 | 144 | 0.57 | - | - | 61 | 0.25 |
| 2000 | 563 | 2.22 | 153 | 0.60 | - | - | 39 | 0.15 |
| 2001 | 550 | 2.13 | 146 | 0.57 | - | - | 48 | 0.19 |
| 2002 | 595 | 2.27 | 150 | 0.57 | - | - | 45 | 0.17 |
| 2003 | 576 | 2.19 | 160 | 0.60 | - | - | 49 | 0.19 |
| 2004 | 618 | 2.30 | 124 | 0.46 | - | - | 39 | 0.14 |
| 2005 | 674 | 2.46 | 182 | 0.66 | - | - | 31 | 0.11 |
| 2006 | 601 | 2.13 | 163 | 0.58 | - | - | 24 | 0.09 |
| 2007 | 657 | 2.31 | 162 | 0.57 | - | - | 35 | 0.12 |
| 2008 | 676 | 2.30 | 172 | 0.60 | - | - | 31 | 0.11 |
| 2009 | 715 | 2.38 | 147 | 0.49 | - | - | 31 | 0.11 |
| 2010 | 712 | 2.31 | 202 | 0.65 | - | - | 29 | 0.10 |
| 2011 | 756 | 2.41 | 147 | 0.46 | - | - | 27 | 0.09 |
| 2012 | 772 | 2.38 | 171 | 0.53 | - | - | 41 | 0.13 |
| 2013 | 759 | 2.35 | 198 | 0.61 | - | - | 39 | 0.12 |
| 2014 | 780 | 2.31 | 221 | 0.65 | - | - | 43 | 0.13 |
| 2015 | 827 | 2.41 | 206 | 0.60 | - | - | 53 | 0.17 |

-Statistic suppressed due to <16 cases in the time interval

^a^ Rates were calculated as number of deaths per 100,000 person-years and age-adjusted to the 2000 U.S. standard population.

eTable 20. Glioblastoma Incidence-Based Mortality Rates^a^ During 1995-2018 Among Glioblastoma Cases Diagnosed During 1983-2018 According to Tumor Size

| Year of death | Tumor size | | | | | | | |
| --- | --- | --- | --- | --- | --- | --- | --- | --- |
|  | ≤3.0 | | >3.0 to ≤5.0 | | >5.0 | | Unknown | |
|  | No. cases | Rate | No. cases | Rate | No. cases | Rate | No. cases | Rate |
| 1995 | 88 | 0.38 | 178 | 0.75 | 94 | 0.40 | 338 | 1.44 |
| 1996 | 108 | 0.46 | 201 | 0.85 | 115 | 0.47 | 312 | 1.32 |
| 1997 | 100 | 0.42 | 171 | 0.71 | 136 | 0.56 | 304 | 1.26 |
| 1998 | 98 | 0.40 | 196 | 0.80 | 130 | 0.53 | 314 | 1.28 |
| 1999 | 108 | 0.43 | 221 | 0.89 | 135 | 0.54 | 302 | 1.21 |
| 2000 | 96 | 0.38 | 225 | 0.89 | 153 | 0.60 | 288 | 1.14 |
| 2001 | 97 | 0.38 | 256 | 0.99 | 149 | 0.58 | 245 | 0.96 |
| 2002 | 138 | 0.53 | 231 | 0.87 | 145 | 0.55 | 279 | 1.07 |
| 2003 | 112 | 0.43 | 252 | 0.96 | 171 | 0.65 | 256 | 0.97 |
| 2004 | 112 | 0.42 | 260 | 0.95 | 173 | 0.65 | 239 | 0.89 |
| 2005 | 164 | 0.60 | 277 | 1.01 | 226 | 0.83 | 224 | 0.81 |
| 2006 | 135 | 0.49 | 268 | 0.95 | 220 | 0.78 | 167 | 0.59 |
| 2007 | 141 | 0.50 | 298 | 1.04 | 235 | 0.82 | 180 | 0.64 |
| 2008 | 135 | 0.47 | 290 | 0.97 | 278 | 0.96 | 176 | 0.60 |
| 2009 | 151 | 0.51 | 335 | 1.12 | 267 | 0.89 | 141 | 0.46 |
| 2010 | 174 | 0.56 | 319 | 1.03 | 264 | 0.85 | 187 | 0.62 |
| 2011 | 173 | 0.55 | 342 | 1.09 | 249 | 0.79 | 166 | 0.54 |
| 2012 | 158 | 0.48 | 368 | 1.13 | 282 | 0.87 | 176 | 0.56 |
| 2013 | 191 | 0.60 | 335 | 1.05 | 293 | 0.89 | 178 | 0.55 |
| 2014 | 193 | 0.56 | 364 | 1.09 | 319 | 0.94 | 168 | 0.50 |
| 2015 | 182 | 0.53 | 401 | 1.17 | 314 | 0.91 | 189 | 0.57 |
| 2016 | 218 | 0.62 | 411 | 1.15 | 356 | 1.03 | 175 | 0.49 |
| 2017 | 207 | 0.57 | 424 | 1.20 | 327 | 0.90 | 144 | 0.41 |
| 2018 | 216 | 0.58 | 396 | 1.07 | 342 | 0.92 | 142 | 0.40 |

^a^ Rates were calculated as number of deaths per 100,000 person-years and age-adjusted to the 2000 U.S. standard population.
